# Transcriptomic profiles conducive to immune-mediated tumor rejection in human breast cancer skin metastases treated with Imiquimod

**DOI:** 10.1038/s41598-019-42784-9

**Published:** 2019-06-12

**Authors:** Mariya Rozenblit, Wouter Hendrickx, Adriana Heguy, Luis Chiriboga, Cynthia Loomis, Karina Ray, Farbod Darvishian, Mikala Egeblad, Sandra Demaria, Francesco M. Marincola, Davide Bedognetti, Sylvia Adams

**Affiliations:** 1 Department of Hematology Oncology, Yale University School of Medicine, New Haven, Connecticut USA; 2 Tumor Biology, Immunology, and Therapy Section, Immunology, Inflammation, and Metabolism Department, Division of Translational Medicine, Sidra Medicine, Doha, Qatar; 3 Department of Pathology, New York University School of Medicine, New York, New York, USA; 4 Genome Technology Center, Division of Advanced Research Technologies, University of New York School of Medicine, New York, New York, USA; 5 Cold Spring Harbor Laboratory, Cold Spring Harbor, New York, New York, USA; 6 Department of Radiation Oncology Weill Cornell Medical College, New York, New York, USA; 7 Refuge Biotechnologies Inc, Menlo Park, CA USA; 8 Laura & Isaac Perlmutter Cancer Center, New York University School of Medicine, New York, New York, USA

**Keywords:** Diagnostic markers, Breast cancer

## Abstract

Imiquimod is a topical toll-like-receptor-7 agonist currently used for treating basal cell carcinoma. Recently, imiquimod has demonstrated tumor regression in melanoma and breast cancer skin metastases. However, the molecular perturbations induced by imiquimod in breast cancer metastases have not been previously characterized. Here, we describe transcriptomic profiles associated with responsiveness to imiquimod in breast cancer skin metastases. Baseline and post-treatment tumor samples from patients treated with imiquimod in a clinical trial were profiled using Nanostring technology. Through an integrative analytic pipeline, we showed that tumors from patients who achieved a durable clinical response displayed a permissive microenvironment, substantiated by the upregulation of transcripts encoding for molecules involved in leukocyte adhesion and migration, cytotoxic functions, and antigen presentation. In responding patients, Imiquimod triggered a strong T-helper-1 (Th-1)/cytotoxic immune response, characterized by the coordinated upregulation of Th-1 chemokines, migration of Th-1 and cytotoxic T cells into the tumor, and activation of immune-effector functions, ultimately mediating tumor destruction. In conclusion, we have shown that topical imiquimod can induce a robust immune response in breast cancer metastases, and this response is more likely to occur in tumors with a pre-activated microenvironment. In this setting, imiquimod could be utilized in combination with other targeted immunotherapies to increase therapeutic efficacy.

## Introduction

Breast cancer is the second cause of death in women and the second most common cancer to metastasize to the skin after melanoma^1^. Treatment of breast cancer skin metastases remains a challenge. Initial management usually consists of resection and radiotherapy but skin metastases often recur and can lead to chest wall ulceration, bleeding, and pain.

Imiquimod is a Toll-like Receptor (TLR)-7 agonist that can activate the innate immune system and shape the ensuing adaptive immune response. Imiquimod induces the production of several pro-inflammatory cytokines such as IFN-*α*, TNF-*α*, IL-1, IL-6, and IL-8 and leads to the recruitment and activation of dendritic cells^2–4^. In basal and squamous cell carcinoma, imiquimod induces infiltration of the tumor with effector T cells, increases production of IFN-γ, granzyme, perforin, and apoptosis^5,6^. Genome-wide transcriptomic analysis of basal cell carcinoma before and after treatment has defined key molecular events induced by imiquimod^7^. Such transcripts are involved in the activation of specific chemokine-chemokine receptor ligand pathways conducive to a T-helper 1 (Th-1) immune response and the induction of immune-effector genes. These pathways are also activated during other forms of immunity-mediated tissue-specific destruction, such allograft rejection^8^, graft-versus-host disease and flares of autoimmunity, and were termed as the Immunologic Constant of Rejection (ICR)^9–11^. The ICR signature was further refined and is now represented by twenty transcripts in four functional categories: CXCR3/CCR5 chemokines (including CXCL9, CXCL10, CCL5), Th1 signaling (including IFNG, IL12B, TBX21, CD8A, STAT1, IRF1, CD8B), effector (including GNLY, PRF1, GZMA, GZMB, GZMH) and immune regulatory functions (including CD274, CTLA4, FOXP3, IDO1, PDCD1)^12–14^. High expression of the ICR has been found to be associated with favorable prognosis in multiple tumor types and responsiveness to immunotherapeutic approaches such as IL-2, adoptive therapy and checkpoint inhibition^10,15–17^.

In melanoma, several case reports have shown regression of cutaneous metastases with imiquimod either alone^18,19^ or in combination with other therapies^20–22^. This tumor regression was associated with an increase in T cell infiltrate and upregulation of ICR genes such as CCL5, CXCL9, CXCL10 important for homing of cytotoxic T cells as well as markers of dendritic cell (CD80, CD86) and T cell activation (CD69)^23^.

Activation of the adaptive immune response has been shown to be associated with better prognosis in breast cancer. An increase in the number of tumor infiltrating lymphocytes is associated with greater likelihood of complete pathological response after chemotherapy across breast cancer subtypes, and reduced risk of relapse and death particularly in triple negative breast cancers^24–31^. Interestingly, most of the prognostic and predictive transcriptomic classifiers described so far in breast cancer include ICR genes. Type I immunity is believed to be especially important for immunotherapy, with CD8+ T cells inducing cytotoxicity and CD4+ T helper cells inducing cytokine secretion and promotion of antigen presentation of tumor^32^. In a recent trial, topical imiquimod in combination with intravenous nab-paclitaxel resulted in an antitumor response of 72% in the treatment of mostly hormone receptor negative breast cancer cutaneous metastases^33^ suggesting that activation of the immune system may play an important role in the treatment of breast cancer skin metastases. Imiquimod may play an even more important role in hormone receptor positive breast cancer, since it is the subtype with the least amount of CD8+ infiltrating T cells^34^.

We have previously shown that topical imiquimod, as a single agent, can induce an antitumor response in 20% of skin metastases of mainly hormone receptor positive breast cancers and can promote an immunogenic tumor microenvironment^35^. An increase in CD4+ and CD8+ infiltrating T cells after treatment was seen in one of the responders but not in the other, and cytokine measurement in tumor supernatant was variable, suggesting the need for a more sensitive assay to characterize the effects of imiquimod on the tumor microenvironment in breast cancer skin metastases. Furthermore, in two additional patients who achieved long-term benefit, an antitumor antigen response was induced by imiquimod (*in-situ* vaccination effect) and subsequently expanded by endocrine therapy leading to durable complete remissions.^36^

Here, by using an integrative analysis we describe transcriptomic profiles associated with responsiveness to imiquimod treatment. This is the first study to characterize the transcriptomic changes induced by imiquimod in breast cancer skin metastases.

## Methods

### Patients

Ten patients were enrolled and treated with imiquimod for eight weeks as previously described^35^. The clinical trial was approved by the New York University Institutional Review Board. All research was performed in accordance with the New York University Institutional Review Board guidelines and regulations, a written informed consent was obtained from all patients. Same-site tumor biopsies were obtained at baseline and after 8 weeks of imiquimod treatment. Paraffin embedded tumor tissue was available from 8/10 patients for this study, as samples from two patients had insufficient quantity for further analysis. Two of the patients had stable disease during the initial study and were found to have a systemic complete clinical response after subsequent treatment with fulvestrant after study completion (including the treated skin metastases). On follow up, these two patients also had disease remission for two years. We initially sought to characterize the tumor microenvironment of these two patients due to their unusual complete response on hormone therapy and long duration of disease remission and labeled these patients as complete responders (CR). An additional patient had a local partial tumor response (greater than 50% tumor shrinkage) after eight weeks of imiquimod treatment and was labeled as a partial responder (PR). Patients with CR and PR were considered as having derived clinical benefit (CB). Five of the eight patients did not have a clinical response and were defined as non-responders (NR).

### Gene Expression

Paraffin blocks were carefully evaluated for tumor content and cut into seven sections of 10 um thickness for RNA extraction. Samples were deparaffinized with xylene and extracted using the RNeasy FFPE kit (Qiagen #73504), according to manufacturer’s instructions. Extracted FFPE RNA quality and quantity were analyzed on an agilent Bioanalyzer 2100 using a nano chip. Smear analysis was performed to determine the percent of RNA greater than 300nt for optimized hybridization according to Nanostring’s protocol. Adjusted inputs for each RNA sample were calculated to input 100 ng of RNA greater than 300nt. RNA was hybridized using the Nanostring nCounter® Human v1.1 PanCancer Immune Profiling Panel (770 transcripts) according to the manufacturer’s protocol. Hybridizations were processed on the nCounter Prep Station, and prepped cartridges were scanned on the Nanostring Digital Analyzer using 280 field of view counts.

### Immunohistochemistry

The evaluation of tumor-infiltrating lymphocytes (TIL) density was performed by Immunohistochemistry (IHC) on paraffin embedded tumor tissues as previously described^35^ and correlated with clinical response. Tumor sections (thickness 4 μm) were deparaffinized and rinsed in distilled water. Heat induced epitope retrieval was carried out in 10 mmol/L citrate buffer. CD3, CD4, CD8 (Ventana Medical Systems), and Forkhead Box Protein P3 antibody (FoxP3, Ebiosciences) antibodies were used. Detection was carried out on a NEXes instrument (Ventana Medical Systems) using the manufacturer’s reagent buffer and detection kits. After washing in distilled water, slides were counterstained with hematoxylin, dehydrated, and mounted with permanent media. Appropriate positive and negative controls were included with the study sections. IHC-positive cells were counted manually in 5 representative high-power fields (HPF, 400), to derive the average number per HPF, by a pathologist blinded to the response.

### Statistical Analysis

Data were normalized using the housekeeping genes present in the panel and by applying negative control subtraction (nSover 2.6 package). Quantile normalization using preprocessCore v1.38 and Log_2_ transformation was applied to the expression matrix. PCA plots were generated using scatterplot3d v0.3. Consensus clustering based on the ICR genes was performed using consensusclusterPlus v1.40 (maxK = 7, 5000 repetitions, Ward D2 as interlinking). Based on such clustering, samples were classified as ICR High, ICR Medium, and ICR Low, with ICR High and ICR Low samples as the highest and lowest expression of the ICR z-score, respectively.

Singe sample gene set enrichment (ssGSEA)^37,38^ using GSVA package v1.24.2 was applied to calculate enrichment scores for leukocyte populations using cell-specific transcripts included in the Nanostring panel (Supplementary Table S1) as well as the enrichment score for the ICR genes. The fold changes of these scores were expressed as the ratio of Anti Log_e_ of the mean enrichment scores and were plotted with their confidence interval using forestplot v1.7.2. Differentially expressed genes (DEGs) or differences in the enrichment scores were calculated by paired or unpaired t-test for the post- vs pre-treatment and CR vs NR analysis respectively using 2-tailed *p* value of 0.05 as significance cut off. Fold change was expressed by the Anti-Log_2_ of the Log_2_ fold change, which is the difference between the mean of the Log_2_ values of the two categories in comparison. Enrichment analysis of the DEGs was performed using Nanostring annotations (immune response categories, Supplementary Table S2) specific for this panel. In all comparisons of response status, only the CR and NR samples were included as these samples represent tumors with two opposite biologic behaviors. PR data were however displayed in the heatmaps and boxplots in comparison with CR and NR samples. Post- vs pre-treatment comparisons were performed by including all samples. Boxplots for single gene expression and ICR score were plotted using ggplot2 v 2.2.1. Dotted heatmaps were used to represent the ssGSEA enrichment z-scores and the percentage of genes with an absolute fold change of more than 1.5 for each immune cell signature. These plots were generated using the ComplexHeatmap v1.14 package. Immunohistochemistry data were compared by Wilcoxon signed-rank test.

## Results

### Patient characteristics

Paired tissues from eight patients with breast cancer skin metastases enrolled on a clinical trial of imiquimod therapy were included in this study. Patient demographics are summarized in Table 1. The majority of the women were post-menopausal (62.5%), all had invasive ductal carcinoma, and the majority of tumors were high grade (87.5%) and hormone receptor positive (87.5%), 50% were HER2 positive. Patients were designated as CR (n = 2), PR (n = 1), and NR (n = 5) as described above.

**Table 1:** Patient characteristics.

ID	Age	Pathology	HR	HER2	Grade	Extracutaneous metastases	Response
1	50	IDC	Pos	Pos	III	Lung	NR
2	44	IDC	Neg	Neg	III	Pleura	NR
3	49	IDC	Pos	Pos	III	None	NR
4	57	IDC	Pos	Neg	III	LN	NR
5	50	IDC	Pos	Pos	III	Adrenal	CR
6	49	IDC	Pos	Neg	II	Lung, Bone	CR
7	57	IDC	Pos	Pos	III	Bone	NR
8	71	IDC	Pos	Neg	III	Bone, LN	PR

ID: identifier, IDC: invasive ductal carcinoma, HR: hormone receptor, HER2: human epidermal growth factor receptor, NR: no response, CR: complete response, PR: partial response. Pos: positive, Neg: negative.

### Transcriptome changes induced by imiquimod treatment and baseline predictive markers

**Figure 1:**
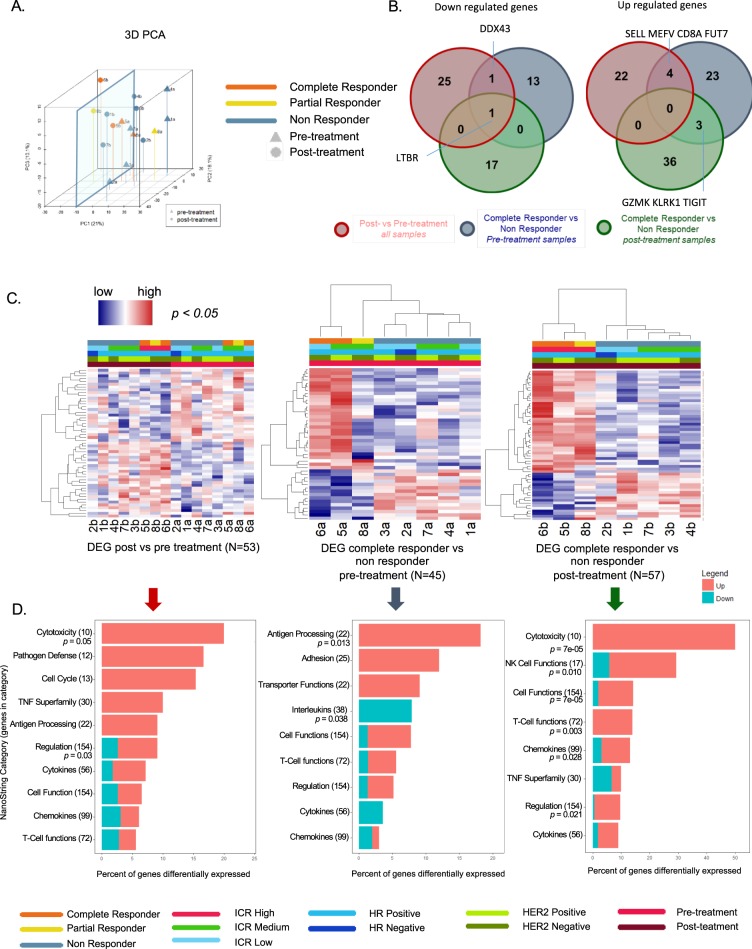
Transcriptomic changes according to treatment and response status. (**A**) Principle component analysis (PCA) based on all transcripts. (**B**) Venn diagrams of DEGs, (separated by down and upregulated, p < 0.05) red circles: paired t-test, post- vs pre-treatment samples; blue circles: unpaired t-test, pre-treatment complete responder (CR) vs pre-treatment non responder (NR), excluding the partial responder (PR) sample from the statistical test; green circles: unpaired t-test, post-treatment complete responder (CR) vs post-treatment non responder (NR), excluding the partial responder sample from the statistical test. (**C**) Heatmaps based on the DEGs in B; samples are ordered according to treatment and ICR score in the left panel, and using hierarchical clustering in the middle and right panel; the PR sample was clustered according to the DEG as in B; genes are ordered according to hierarchical clustering in all panels; Hormone receptor (HR) status, HER2 receptor (HER2) status and ICR classification are shown. For the ICR classification, samples were clustered using consensus clustering based on 20 ICR genes in 3 groups with low (ICR low), intermediate (ICR medium), and high (ICR high) expression of the ICR genes (see methods). (**D**) Top enriched Nanostring pathways (Immune Response Categories).

Principal component analysis (PCA) based on all the transcripts is shown in Fig. 1A. Using a PC1 arbitrary cut-off of −10 3/3 tumors from patients responding to the treatment but only 1/5 tumors from non-responding patients were found to cluster. The number of overlapping genes among the comparisons used for the detection of the DEGs (e.g., post vs pre-treatment, pre-treatment CR vs NR, and post-treatment CR vs NR) are shown in Fig. 1B. Comparing all patients post- versus pre-treatment, 53 DEGs were identified (Table 2, Fig. 1B,C). Several genes related to innate immunity were upregulated in the post-treatment group. These included genes related to lymphocyte activation (SH2D1A, SLAMF1), lymphocyte adhesion (SELL), cytolysis (PRF1), NK cell activation (KLRC1), T cell (CD3G, CD347) and cytotoxic T cell (CD8A) function, and antigen presentation (HLA-C). Although some genes were downregulated, overall, pathway enrichment analysis revealed a positive modulation of the immune response underlined by the activation of cytotoxic mechanisms (Fig. 1D and Supplementary Table S3). Downregulated genes included several MAPK-related genes (MAP2K4, MAPK1, MAPK11 and MAP3K5), which are also involved in oncogenic signaling, and genes related to IL-17/Th-17 and IL-13 signaling (Table 2 and Supplementary Table S3).

**Table 2:** DEG post vs pre-treatment.

Gene	p value	q value	FC (Post vs Pre)	Annotation
SELL	0.0111	0.725	2.212	CD molecules, Regulation of immune response
PRF1	0.0097	0.725	2.119	Adaptive immune response, Cell Type specific, Cytotoxicity, Defense response to tumor cell, Defense response to virus
SH2D1A	0.0259	0.725	2.099	Humoral immune response
TNFRSF13B	0.0152	0.725	1.987	CD molecules, Chemokines and receptors, TNF superfamily members and their receptors
CD3G	0.0326	0.725	1.891	Adaptive immune response, Cell Type specific, CD molecules, Regulation of immune response, T-cell activation
TNFRSF9	0.048	0.725	1.860	CD molecules, TNF superfamily members and their receptors
IRF7	0.0182	0.725	1.813	Innate immune response
CD38	0.0292	0.725	1.733	Adaptive immune response, Cell Type specific, CD molecules, Response to drug, Positive regulation of B-cell proliferation
CD247	0.0116	0.725	1.583	CD molecules, Regulation of immune response
IL1R2	0.0436	0.725	1.551	CD molecules, Cytokines and receptors
KLRC1	0.0035	0.725	1.547	CD molecules, NK cell functions, Regulation of immune response
CDKN1A	0.0443	0.725	1.543	Cell cycle checkpoint and cell cycle arrest, Regulation of cell cycle, Senescence pathway, Senescence initiators interferon related
MEFV	0.0089	0.725	1.543	Innate immune response
CD8A	0.0059	0.725	1.537	Adaptive immune response, Antigen processing and presentation, Cell Type specific, CD molecules, Defense response to virus, T-cell activation, T-cell differentiation
CD6	0.0349	0.725	1.513	Adaptive immune response, Basic cell functions, Cell Type specific, CD molecules
NOD2	0.0322	0.725	1.509	Innate immune response, Cytokines and receptors
FUT7	0.0485	0.725	1.505	Leukocyte migration
SLAMF1	0.0039	0.725	1.477	CD molecules, Adaptive immune response
TNFSF10	0.038	0.725	1.456	CD molecules, Co-Regulators of autophagy and apoptosis/cell cycle, Cytokines and receptors, TNF superfamily members and their receptors
ITGAL	0.0276	0.725	1.452	Adhesion, CD molecules, Regulation of immune response
NLRC5	0.0359	0.725	1.422	Innate immune response
HLA-C	0.0173	0.725	1.397	Adaptive immune response, Antigen processing and presentation, Cytotoxicity, Regulation of immune response
STAT2	0.0331	0.725	1.379	Cytokines and receptors, Transcription factors, Transcriptional regulators
MME	0.0142	0.725	1.299	Basic cell functions, Cell Type specific, CD molecules
CD83	0.0138	0.725	1.273	CD molecules, Humoral immune response
CMKLR1	0.0412	0.725	1.198	Chemokines and receptors
EWSR1	0.02	0.725	0.857	Basic cell functions, Cell Type specific
MAP2K2	0.0088	0.725	0.837	Innate immune response
DDX43	0.0092	0.725	0.792	
MICA	0.0182	0.725	0.791	Immune response to tumor cell, Regulation of immune response
MAP3K5	0.0008	0.328	0.788	Innate immune response
CD200	0.0283	0.725	0.782	CD molecules, Regulation of immune response
LTBR	0.0061	0.705	0.780	Chemokines and receptors
PVR	0.0037	0.328	0.767	CD molecules, Regulation of immune response
C5	0.044	0.725	0.729	Complement pathway, Innate immune response
TAB1	0.0277	0.725	0.726	Innate immune response
MAPK11	0.0325	0.725	0.720	Innate immune response
IL17B	0.0201	0.725	0.709	Chemokines and receptors, Interleukins
MAPK1	0.0021	0.705	0.705	Innate immune response
CD164	0.0427	0.725	0.702	CD molecules
NFKBIA	0.043	0.725	0.695	Innate immune response
ILF3	0.0018	0.517	0.675	Chemokines and receptors
IL13RA1	0.0167	0.725	0.627	CD molecules, Cytokines and receptors, Th2 orientation
MST1R	0.0258	0.725	0.621	CD molecules, Innate immune response
MAP2K4	0.0309	0.725	0.618	Innate immune response
APOE	0.0236	0.725	0.594	Cell Type specific, Lipid transporter activity
RPS6	0.043	0.725	0.586	T-cell proliferation
CD9	0.0178	0.725	0.571	CD molecules
TXNIP	0.0434	0.725	0.559	Innate immune response
F13A1	0.0372	0.725	0.507	Basic cell functions, Cell Type specific
DUSP4	0.0337	0.725	0.501	Innate immune response
CMA1	0.0413	0.725	0.421	Cell Type specific, Regulation of inflammatory response
FCER1A	0.0152	0.725	0.336	Inflammatory response

53 Significant (p < 0.05) DEGs comparing nanostring immune gene expression between post- and pre- treatment samples using paired t-test including non responder (NR), complete responder (CR) and partial responder (PR) samples. Ordered from high to low fold change (Fc = 2^(mean log2 post treatment expression − mean log2 pre treatment expression)).

In baseline samples, prior to treatment with imiquimod, 45 genes were differentially expressed in CR vs NR samples (Table 3, Fig. 1B). Interestingly, the PR clustered between CR and NR (Fig. 1C). Upregulated genes included leukocyte adhesion and migration molecules (ITGAX, ITGAM, ITGB2, and FUY7), chemokine receptor (CX3CR1), T-cell cytotoxic markers (CD8A, and GZMK), and genes associated with antigen presentation (HLA-DMA, HLA-DBP1, and HLA-DPA1). Enrichment analysis corroborated the existence of a permissive microenvironment in lesions from patients with clinical benefit from imiquimod, substantiated by the activation of the antigen processing pathway, which was accompanied by the downregulation of some cytokines such as IFN lambda 1 (IFNL1) IL34, and IL23, which is involved in Th-17 signaling (Fig. 1D).

**Table 3:** DEG CR vs NR (pre-treatment).

Gene	p value	q value	FC (CR vs NR)	Annotation
CFB	0.00341	0.256	7.534	Innate immune response
DUSP4	0.00981	0.368	7.489	Innate immune response
MPPED1	0.00008	0.047	4.988	Basic cell functions, Cell Type specific
HLA-DPA1	0.00137	0.163	3.120	Adaptive immune response, Antigen processing and presentation
ITGAX	0.03974	0.638	2.971	Adhesion, CD molecules, Innate immune response
C3	0.01982	0.499	2.720	Innate immune response, Regulation of immune response
HLA-DPB1	0.00069	0.137	2.561	Adaptive immune response, Antigen processing and presentation
PTPRC	0.04822	0.638	2.535	B-cell proliferation, CD molecules, T-cell differentiation
CX3CR1	0.0117	0.368	2.526	Adaptive immune response, Chemokines and receptors, Microglial cell activation
FUT7	0.00345	0.256	2.488	Leukocyte migration
CTSS	0.01084	0.368	2.440	Adaptive immune response
MSR1	0.03954	0.638	2.267	Basic cell functions, Cell Type specific, CD molecules
FCGR2B	0.04684	0.638	2.132	CD molecules, Regulation of immune response
MEFV	0.00276	0.256	2.085	Innate immune response
CD68	0.02311	0.528	2.074	Basic cell functions, Cell Type specific, CD molecules
ITGAM	0.00035	0.104	2.064	Adhesion, CD molecules, Innate immune response, Receptors involved in phagocytosis
CHIT1	0.04168	0.638	2.006	Basic cell functions, Cell Type specific
GZMK	0.00124	0.163	1.933	Adaptive immune response, Basic cell functions, Cytotoxicity, Innate immune response
ITGB2	0.02745	0.604	1.859	Adhesion, CD molecules, Regulation of immune response
CD53	0.02249	0.528	1.819	CD molecules, Adaptive immune response
CRP	0.04046	0.638	1.793	Acute-phase response, Humoral immune response, Inflammatory response, Innate immune response, Receptors involved in phagocytosis
HLA-DMA	0.04879	0.638	1.687	Adaptive immune response, Antigen processing and presentation, Positive regulation of immune response
CD8A	0.03163	0.638	1.548	Adaptive immune response, Antigen processing and presentation, Cell Type specific, CD molecules, Defense response to virus, T-cell activation, T-cell differentiation
HAVCR2	0.0361	0.638	1.541	Basic cell functions
DUSP6	0.02018	0.499	1.507	Innate immune response
KLRK1	0.01208	0.368	1.478	CD molecules, NK cell functions, Regulation of immune response
LY86	0.00539	0.294	1.466	Innate immune response, Humoral immune response
SPACA3	0.01239	0.368	1.456	
TIGIT	0.04384	0.638	1.399	Adaptive immune response, T-cell activation
SELL	0.04335	0.638	1.285	CD molecules, Regulation of immune response
MARCO	0.04824	0.638	0.812	Cell Type specific, Innate immune response
IL1RAP	0.03197	0.638	0.745	Innate immune response
MYD88	0.0474	0.638	0.716	Innate immune response, Toll-like receptor
LTBR	0.01324	0.374	0.702	Chemokines and receptors
DDX43	0.01743	0.470	0.700	
IL2RA	0.04836	0.638	0.661	Adaptive immune response, CD molecules, Inflammatory response to antigenic stimulus, Negative regulation of immune response
PLA2G1B	0.01173	0.368	0.579	Positive regulation of immune response
IL23A	0.00561	0.294	0.566	Innate immune response, Interleukins
PSMD7	0.00675	0.308	0.564	CD molecules
IL12RB2	0.04282	0.638	0.550	Adaptive immune response, Cell Type specific, Cytokines and receptors, Th1 orientation
S100B	0.04302	0.638	0.529	Innate immune response
IFNL1	0.00504	0.294	0.270	Cytokines and receptors, Interleukins
IL34	0.00839	0.356	0.248	Innate immune response, Interleukins
HSD11B1	0.00595	0.294	0.177	Basic cell functions, Cell Type specific
PPBP	0.03365	0.638	0.115	Chemokines and receptors

45 Significant (p < 0.05) DEGs comparing nanostring immune gene expression in pre-treatment samples between CR and NR using unpaired t-test (PR sample was excluded). Ordered from high to low fold change (Fc = 2^(mean log2 CR − mean log2 NR expression)).

After imiquimod treatment, CR versus NR differed in 57 DEGs (Table 4, Fig. 1B). In this analysis, the PR clustered together with the CR. CR patients had higher levels of several immune genes (Fig. 1C), including chemokines and chemokine receptors (CXCL13, CXCL9, CCL5, CCR5, CXCR3, CCR7, CXCR4), markers of lymphocyte activation (LAG3, TXK), NK cells (KLRC2, KLRD1, NCR1), neutrophils (LCN2), and markers of effector function (GNLY, GZMB, GZMK, GZMA), and innate immunity (TNFRSF1B, IL7R). S100A7, which is involved in the regulation of cell cycle progression and proliferation, was also over-expressed. The top enriched pathways in the CR group included NK and T-cell functions, chemokines and (immune) regulation (Fig. 1D).

**Table 4:** DEG CR vs NR (post-treatment).

Gene	p value	q value	FC (CR vs NR)	Annotation
S100A7	0.02973	0.297	14.094	Innate immune response
CXCL9	0.00433	0.245	8.161	Adaptive immune response, Chemokines and receptors, Regulation of inflammatory response, Th1 orientation
LAG3	0.04199	0.297	6.555	Adaptive immune response, CD molecules, Negative regulation of immune response, T-cell activation
CCL5	0.00923	0.245	5.442	Cytokines and receptors, Chronic inflammatory response, Chemokines and receptors, Acute-phase response, Inflammatory response, Innate immune response
IL7R	0.0062	0.245	5.370	Adaptive immune response, CD molecules, Cytokines and receptors
GNLY	0.02575	0.294	4.774	Adaptive immune response, Cell Type specific, Cytotoxicity, Defense response to bacterium, Defense response to fungus
LCN2	0.00015	0.028	4.756	Innate immune response
KLRC2	0.00007	0.026	4.543	CD molecules, NK cell functions
CXCL13	0.02195	0.294	4.526	Adaptive immune response, Cell Type specific, Chronic inflammatory response, Humoral immune response, Chemokines and receptors
SPINK5	0.02524	0.294	4.480	Negative regulation of immune response
CXCR4	0.00936	0.245	4.411	Adaptive immune response, CD molecules, Chemokines and receptors, Co-Regulators of autophagy and apoptosis/cell cycle, T-cell polarization
GZMB	0.02651	0.294	3.997	Adaptive immune response, Basic cell functions, Cell Type specific, Cytotoxicity
RUNX3	0.00994	0.245	3.693	Transcription factors
HLA-G	0.02265	0.294	3.657	Regulation of immune response
IL2RG	0.02719	0.294	3.419	Adaptive immune response, CD molecules, Chemokines and receptors
CCR5	0.00204	0.245	3.309	CD molecules, Cytokines and receptors, T-cell polarization
KLRK1	0.00996	0.245	3.262	CD molecules, NK cell functions, Regulation of immune response
TNFRSF1B	0.03119	0.297	2.966	CD molecules, Chemokines and receptors, TNF superfamily members and their receptors
KLRD1	0.03172	0.297	2.874	Cell Type specific, NK cell functions, Regulation of immune response
HLA-A	0.00419	0.245	2.810	Adaptive immune response, Antigen processing and presentation, Cytotoxicity, Regulation of immune response
CCR7	0.02747	0.294	2.791	CD molecules, Chemokines and receptors, Humoral immune response, Regulation of inflammatory response
CSF2RB	0.01803	0.294	2.693	CD molecules, Chemokines and receptors, Adaptive immune response
TXK	0.0334	0.297	2.662	Adaptive immune response
STAT4	0.0112	0.246	2.413	Adaptive immune response, Cytokines and receptors, Cell Type specific, Transcription factors, Transcriptional regulators, Th1 orientation
ITK	0.00646	0.245	2.281	Adaptive immune response
IL18RAP	0.02112	0.294	2.195	CD molecules, Innate immune response, Th1 orientation
CD48	0.00366	0.245	2.041	CD molecules
GZMA	0.00561	0.245	2.016	Adaptive immune response, Basic cell functions, Cell Type specific, Cytotoxicity
GZMK	0.0156	0.292	1.999	Adaptive immune response, Basic cell functions, Cytotoxicity, Innate immune response
IL12RB2	0.04061	0.297	1.943	Adaptive immune response, Cell Type specific, Cytokines and receptors, Th1 orientation
LCK	0.04913	0.298	1.943	Regulators of T-cell activation
INPP5D	0.04684	0.298	1.918	Negative regulation of immune response
CXCR3	0.04053	0.297	1.792	Adaptive immune response, CD molecules, Chemokines and receptors, T-cell polarization
CD27	0.04115	0.297	1.736	B-cell differentiation, CD molecules
SMPD3	0.02911	0.297	1.720	Basic cell functions, Cell Type specific
TIGIT	0.02512	0.294	1.720	Adaptive immune response, T-cell activation
CASP8	0.02123	0.294	1.636	Innate immune response
NCR1	0.04159	0.297	1.449	Basic cell functions, Cell Type specific, CD molecules, NK cell functions
ICAM2	0.01182	0.246	1.328	Adhesion, CD molecules, Regulation of immune response
LTBR	0.04521	0.298	0.735	Chemokines and receptors
TNFRSF1A	0.03391	0.297	0.634	CD molecules, Chemokines and receptors, TNF superfamily members and their receptors
TLR7	0.03379	0.297	0.633	Microglial cell activation, Innate immune response, Toll-like receptor
PTGS2	0.03499	0.297	0.601	Acute-phase response, Cytokines and receptors
CD63	0.03564	0.297	0.583	CD molecules
IRAK1	0.00784	0.245	0.577	Innate immune response
KIR_Activating_Subgroup_2	0.04908	0.298	0.541	
CTSG	0.03915	0.297	0.531	Cell Type specific, Defense response to fungus, Positive regulation of immune response
KIR3DL3	0.0223	0.294	0.521	CD molecules, NK cell functions
GPI	0.01482	0.292	0.509	Humoral immune response
HRAS	0.03918	0.297	0.501	Senescence pathway
TREM2	0.02713	0.294	0.477	Humoral immune response
MNX1	0.02152	0.294	0.452	Humoral immune response
USP9Y	0.01048	0.245	0.417	Basic cell functions, Cell Type specific
TNFRSF11B	0.04159	0.297	0.387	TNF superfamily members and their receptors
CD1A	0.00694	0.245	0.322	Basic cell functions, Cell Type specific, CD molecules
FOXJ1	0.02742	0.294	0.290	Cell Type specific, Humoral immune response
CCL28	0.03893	0.297	0.182	Chemokines and receptors

57 Significant (p < 0.05) DEGs comparing nanostring immune gene expression in post treatment samples between CR and NR using unpaired t-test (PR sample was excluded). Ordered from high to low fold change (Fc = 2^(mean log2 CR − mean log2 NR expression)).

Applying multiple hypothesis correction of a 0.25 false discovery rate (q values provided in Tables 2, 3 and 4), yielded 5 genes in the pre-treatment comparison (MPPED1, ITGAM, HLA-DPB1, HLA-DPA1, and GZMK, all upregulated in the CR vs NR samples) and 18 genes in the post-treatment comparison (ICAM2, GZMA, CD48, ITK, STAT4, HLA-A, KLRK1, RUNX3, CXCR4, KLRC2, LCN2, IL7R, CCL5, CXCL9, and CCR5, upregulated in CR vs NR samples and CD1A, USP9Y, and IRAK1, upregulated in NR vs CR samples). The majority of the transcripts upregulated in CR samples relate to chemoattraction, antigen presentation (pre-treatment comparison), and cytotoxic mechanisms (post-treatment comparison). No genes passed the 0.25 false discovery rate in the pre vs post-treatment comparison likely due to the dilution of the signal caused by the hypo-responsive samples.

### Responders to imiquimod treatment had higher levels of T cells and cytotoxic cells

**Figure 2:**
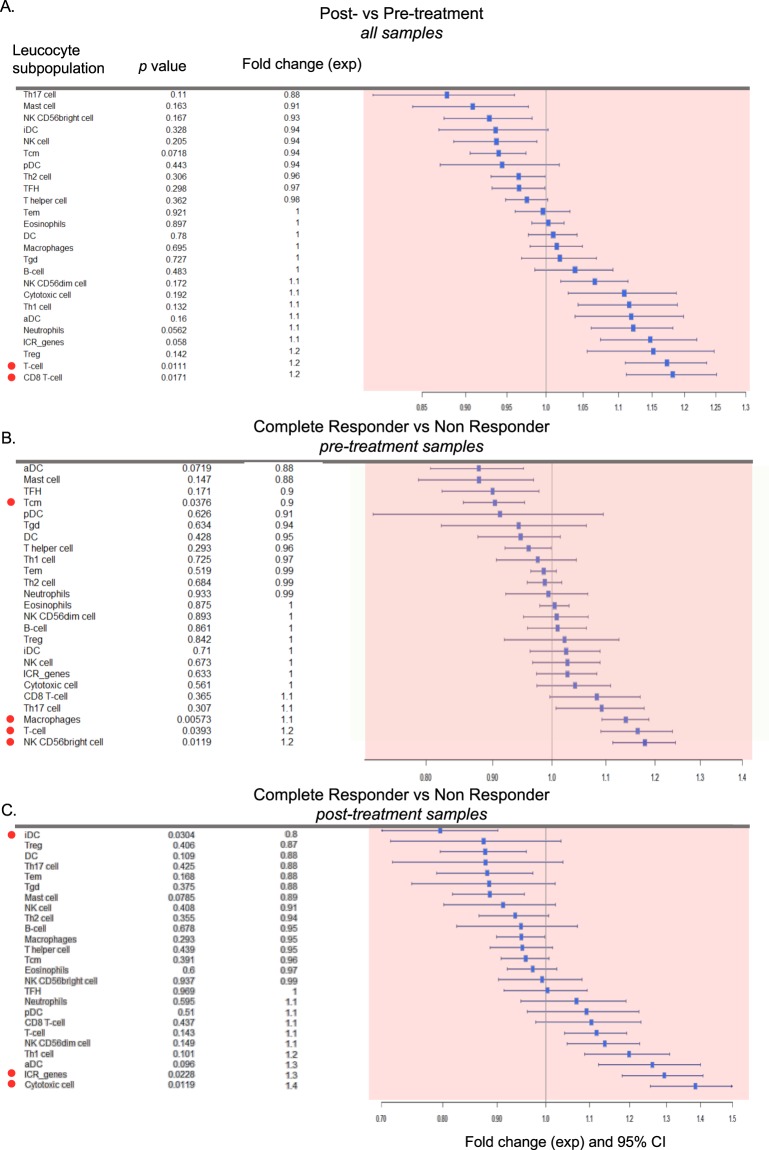
Variation in leukocyte subpopulations according to treatment and response status as estimated by transcriptomic analysis. (**A–C**) Forest plots based on fold changes of leukocyte subpopulation enrichment score. The enrichment score for each leukocyte subpopulation is calculated by single sample gene set enrichment analysis (ssGSEA). Fold changes (FC) are calculated as the ratio of Anti Log_e_ of the enrichment scores. FC > 1 consists of increased enrichment score in post- vs pre-treatment sample in (**A**), increased enrichment score in pre-treatment CR vs pre-treatment NR samples in (**B**), and increased enrichment score in post-treatment complete responder vs post-treatment non responder samples (**C**). *p-*values are from paired (**A**) and unpaired (**B,C**) t-test. The PR was not included in the analysis. Red dots highlight the significant findings *(p* < 0.05, two-tailed).

Single sample gene set enrichment analysis (ssGSEA) was used to estimate the variation in leukocyte subpopulations in each sample. Post- vs pre-treatment comparison revealed that overall, imiquimod consistently enhanced T cell (p = 0.011) and cytotoxic T cell (p = 0.017) infiltration (Fig. 2A). Before treatment, CR patients had a lower level of T central-memory (Tcm) cells (p = 0.038) and a higher level of macrophages (p = 0.0057), T cell (p = 0.039) and NKCD56 bright cells (p = 0.012) as compared to NR patients. The relative depletion of Tcm cells could be the result of a proportional enrichment of activated T-cell after treatment. Thus, CR patients displayed a baseline pre-activated immune microenvironment (Fig. 2B).

**Figure 3:**
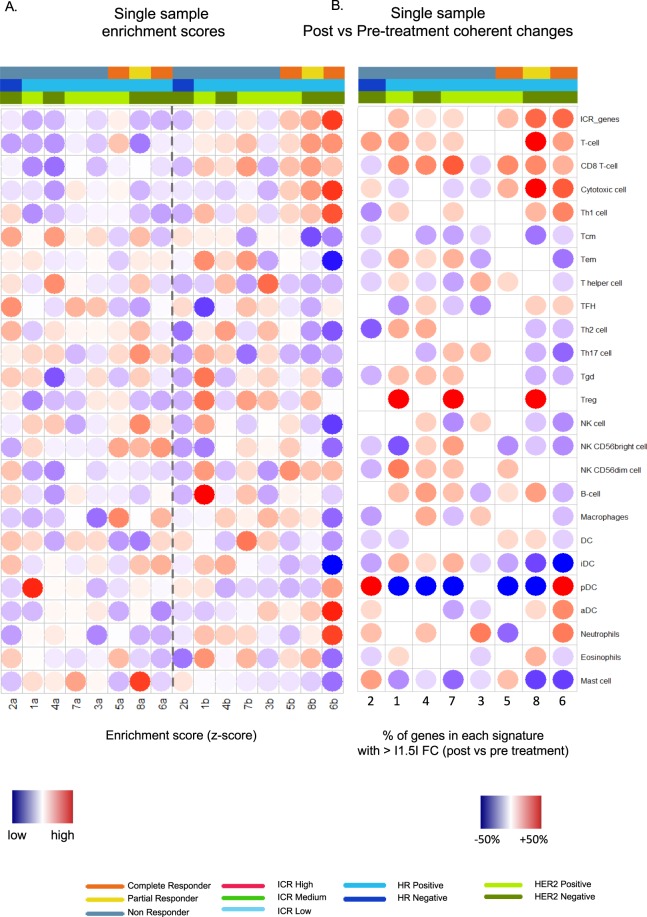
Single sample enrichment scores and consistent changes induced by imiquimod. (**A**) Dotted heatmaps representing the single sample gene set enrichment analysis (ssGSEA) of immune signatures with at least 3 genes present in the nanostring data, with labels for response (NR, PR, CR), treatment (post and pre), hormone receptor (HR) status, HER2 receptor (HER2) status and ICR classification, samples are ordered according treatment and ICR score. (**B**) Matching dotted heatmap representing the percentage of genes in the signature up or downregulated more the 1.5 fold between post and pre-treatment, with inversely regulated genes canceling each other out, cut-off at 15%.

In CR patients, there was a strong activation of the cytotoxic response (p = 0.012) accompanied by upregulation of the ICR genes (Fig. 2C) (p = 0.023) and by a switch from immature dendritic cells (iDC) (p = 0.0228) to activated dendritic cells (aDCs) (p = 0.096) in post-treatment compared to baseline biopsy. At the individual sample level, the magnitude of the cytotoxic response was maximal in responding patients, with similar levels among the two CR and the PR (Fig. 3A,B).

A nonsignificant trend for higher CD3, CD8, CD4, and FOXP3 positive cells, as evaluated by immunohistochemistry, was observed in CR vs NR pre-treatment samples and was also present after treatment. However, among the responding patients, IHC evaluation detected increase of TILs (consistent across all the markers) only in the PR patient (Supplementary Figure 1), who had a local response at the time of the biopsy. This observation suggests that molecular profiling might be more sensitive than IHC in detecting changes in the functional orientations of the tumor microenvironment preceding the clinical response.

### Responders to imiquimod treatment displayed consistent upregulation of the ICR score

**Figure 4:**
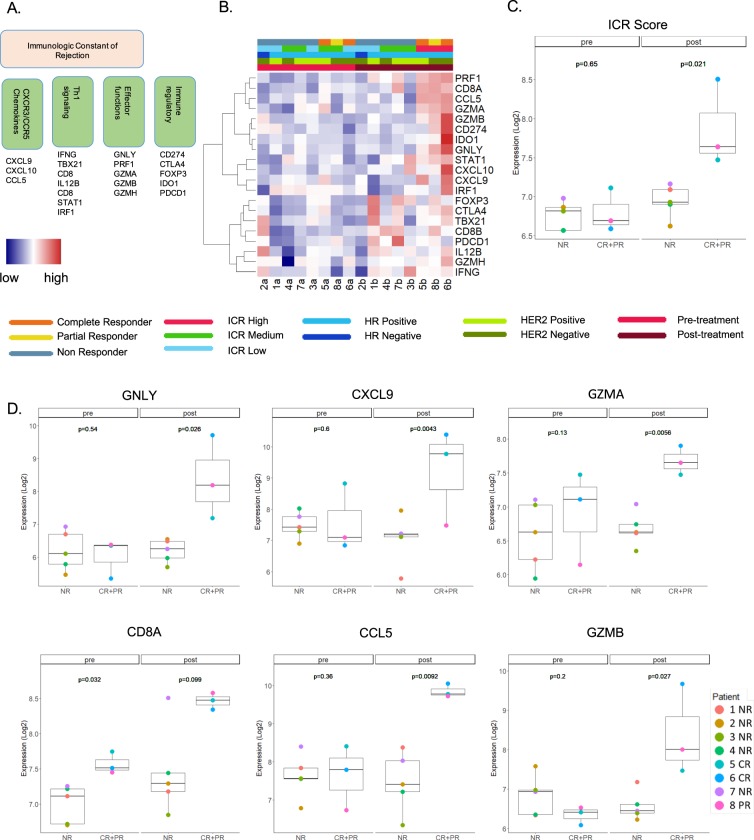
ICR gene expression according to treatment and response status. (**A**) Schematic representation of ICR genes divided by functional categories. (**B**) Heatmap based on ICR gene expression, with labels for response (NR, PR and CR), treatment (post and pre), hormone receptor (HR) status, HER2 receptor (HER2) status and ICR classification. (**C**) Boxplot of the ICR score expressed as the mean log2 expression of the ICR genes according to treatment and response status. (**D**) Boxplots of representative ICR genes: CD8A (Th1 signaling), CXCL9 and CCL5 (CXCR3 and CCR5 signaling), GZMA, GZMB and GNLY (effector functions); *p* values displayed here were calculated using unpaired t-test including only CR and NR. PR data was displayed in the boxplots for comparison with the other two categories.

We analyzed the immunologic constant of rejection (ICR) score (ie., the mean log2 expression of the ICR genes, Fig. 4A,B) across samples, and compared its expression in CR versus NR, pre- and post-treatment. The ICR score was significantly higher in the post-treatment CR vs NR samples (p = 0.021) while it did not differ among the two categories at baseline (Fig. 4C). Interestingly, the PR sample clustered with the CR samples. The ICR genes with significantly higher levels in CR vs NR samples after treatment are shown in Fig. 4D. Most of the ICR genes had similar and consistent trends (Supplementary Figure 2). An exception was represented by FOXP3, which displayed a non significant lower level in post-treatment CR vs NR samples.

## Discussion

This is the first study to characterize the transcriptional changes induced by imiquimod in breast cancer skin metastases. The CR samples showed a pre-activated immune microenvironment at baseline, substantiated by the enrichment of genes related to antigen processing and a proportional enrichment of T cells, NKCD56 bright cells and macrophages. Macrophages have often been shown to be a negative prognostic factor, although M1 macrophages, which sustain anti-tumor immune response, have been associated with favorable prognosis^39,40^. However, coordinated activation of the cytotoxic response was observed only after treatment, suggesting that despite a permissive microenvironment observed at baseline, tumor destruction in responding lesions requires a strong switch from chronic to acute inflammation, which is captured by the ICR genes.

Only a limited proportion of DEG overlaps between pre-treatment CR vs NR, post-treatment CR vs NR, and post-vs pre-treatment lesions. The lack of overlap among DEG post- vs pre-treatment with the ones detected in the post-treatment CR vs NR comparison might be the result of the dilution of the signal in the post-vs pre-treatment comparison due to the inclusion of the NR samples. The differences observed in pre- and post-treatment CR vs NR samples might be the consequence of a differential functional activation of the immune response at these time points. Baseline CR samples are dominated by a sub-inflamed microenvironment typified by the upregulation of antigen-processing related genes, while post-treatment differences are characterized by the coordinated activation of cytotoxic mechanisms conducive to immune-mediated rejection. Nevertheless, 40% (3/7) of the upregulated genes overlapping between at least two comparisons reflect the activation of a cytotoxic response (CD8A, GZMK, KLRK1) while the remaining ones are related to immune modulation. This observation suggests that the full activation of a pre-existing, yet incomplete, cytotoxic response is critical to induce immune-mediated tumor rejection in this setting.

Clinical response to imiquimod treatment was associated with a higher ICR score validating the ICR hypothesis in metastatic breast cancer. The ICR consists of Th-1 signaling genes, CXCR3 and CCR5 chemokines, effector function genes, and immune regulatory genes. High expression levels of these genes have been correlated with better prognosis in melanoma, ovarian, colorectal, hepatocellular, lung, as well as breast cancer^10,12,14^, and in lesions more likely to respond to immunotherapy^10,15–17^. Recently, Ayers *et al*. identified and validated an immune gene signature correlating with clinical benefit to PD1 blockade^41^. This 18-gene signature includes IFN-γ–inducible genes (STAT1, HLA-E), transcripts related to antigen presentation (HLA-DQA1, HLA-DRB1, PSMB10, CMKLR1), chemokines and chemokine ligands (CCL5, CXCL9, CXCR6), cytotoxic activity (CD8A, NKG7), and regulatory functions (TIGIT, LAG3, CD274 (PDLI), CD276, and PDCD1LG2(PDL2)) and as such highly overlaps with the ICR genes^42^.

LAG-3 was identified as one of the DEGs upregulated with a 6.6 fold change in the CR group post-treatment. LAG-3 is a receptor expressed by activated T lymphocytes and is considered a marker of T cell exhaustion leading to reduced effector function and expression of inhibitory receptors^43^. Blockade of LAG-3 is hypothesized to synergize with other immune checkpoint inhibitors such as anti-PD-1^44^. Phase II data of imiquimod with nab-paclitaxel also showed that patients with a poor clinical response had higher circulating levels of PD-1+ T cells suggesting that combination therapy with anti-PD-1 and/or anti-LAG-3 may increase treatment responses^33^. Previous clinical trial data, of mostly estrogen receptor positive metastatic breast cancer, suggest that when combined with chemotherapy, a LAG-3 inhibitor in metastatic breast cancer can increase the number of activated monocytes, dendritic cells, NK cells and CD8 T cells with promising response rates^45^. Our data suggests that LAG-3 may play an important role in breast cancer skin metastases as well and LAG-3 inhibition and PD-1 inhibition should be considered in combination with imiquimod as possible treatment modalities.

Interestingly, the PR sample had the same immune gene expression signature post-treatment (clustering together with the 2 CR samples) as the complete responders (Fig. 1C). Our subsequent long-term analyses of tumor antigen-specific immune responses point to the important role of imiquimod treatment inducing the adaptive response, which then got augmented during endocrine therapy in both patients as they continued to derive clinical benefit and entered a long-lasting CR^36^. This likely explains the unexpected and durable complete response from fulvestrant since a previous large phase 3 study of fulvestrant monotherapy demonstrated that a complete response is very rare^46^. This further suggests that the genes upregulated by imiquimod are associated with long term immunogenic effects which can improve the efficacy of subsequent therapies. Whereas upregulation of ICR genes has been previously correlated with better response to chemotherapy and trastuzumab^29,47^, our data suggests that activation of these pathways may also improve response to subsequent hormonal therapy.

Limitations of our study are the small number of patients and the short duration of treatment. The delayed CR in two patients who displayed immune activation within the metastases after eight weeks of treatment with imiquimod suggests the possibility that a longer treatment might increase response rates. Genes differentially expressed detected by univariate analysis are subject to false discovery. The possibility to incur into a Type I error (i.e., the rejection of a true null hypothesis) is partially attenuated by the use of the integrative pipeline presented here. By combining deconvolution and enrichment analyses, more permissive cut-off p values can be used to detect biological relevant phenomena. Nevertheless, this dataset should be considered as a discovery set, and further validation using orthogonal platforms as deep phenotyping and proteomic approaches are warranted.

The strength of this study is the use of gene array analysis in paired samples derived from a prospective clinical trial with long-term follow-up of patients. This sensitive technique for characterizing immune changes induced by imiquimod treatment was able to detect at 8 weeks intratumoral changes associated with not only immediate but also future clinical benefit, which could not be derived from histologic analysis^35^.

Metastatic breast cancer, especially with cutaneous metastases, remains very difficult to treat. Long-term clinical response to therapy in metastatic cancer is rare. Promoting immunogenicity in the tumor microenvironment may enhance efficacy of other therapies such as chemotherapy, endocrine therapy, targeted therapies or even newer immunotherapeutics. Imiquimod has been shown to be a potent activator of the innate immune system, which then supports adaptive immune response in cancer, and we demonstrate that this is the case in metastatic breast cancer as well. Encouragingly, although hormone receptor positive breast cancer appears to be the least immunogenic type of breast cancer^34^, we have shown that imiquimod can induce a robust anti-tumor immune response leading to clinical response in the three patients with hormone receptor positive disease described here. This is in line with a recent report of successful immunotherapy in a patient with metastatic hormone receptor positive breast cancer^48^. Future trials are needed to investigate the use of imiquimod in combination with other therapies.

## Supplementary information

Supplementary Information

Supplementary File 1

## Data Availability

The normalized Nanostring data used in the current study is available as Supplementary File 1.
