# Supplementary material for: Transcriptomic profiles conducive to immune-mediated tumor rejection in human breast cancer skin metastases treated with Imiquimod
Source: Sci Rep. 2019 Jun 12;9:8572. doi: 10.1038/s41598-019-42784-9 (PMC6561945; doi:10.1038/s41598-019-42784-9)
Supplement: Supplementary file 1 — Supplementary Information [file 41598_2019_42784_MOESM1_ESM.docx]

**Transcriptomic profiles conducive to immune-mediated tumor rejection in human breast cancer skin metastases treated with Imiquimod**

Mariya Rozenblit^1^, Wouter Hendrickx^2^, Adriana Heguy^3,4^, Luis Chiriboga^3^, Cynthia Loomis^3^, Karina Ray^3^, Farbod Darvishian^3^, Mikala Egeblad^5^, Sandra Demaria^6^ ,Francesco Marincola^7^,Davide Bedognetti*^2^, Sylvia Adams*^8^

1 Department of Hematology Oncology, Yale University School of Medicine, New Haven, Connecticut, USA

2 Tumor Biology, Immunology, and Therapy Section, Immunology, Inflammation, and Metabolism Department, Division of Translational Medicine, Sidra Medicine, Doha, Qatar, dbedognetti@sidra.org

3 Department of Pathology, New York University School of Medicine, New York, New York, USA

4 Genome Technology Center, Division of Advanced Research Technologies, University of New York School of Medicine, New York, New York, USA

5 Cold Spring Harbor Laboratory, Cold Spring Harbor, New York, New York, USA

6 Department of Radiation Oncology Weill Cornell Medical College, New York New York USA

7 Refuge Biotechnologies Inc, Menlo Park, CA, USA

8 Laura & Isaac Perlmutter Cancer Center, New York University School of Medicine, New York, New York, Sylvia.Adams@nyumc.org

*DB and SA are co-senior and corresponding authors

**Supplementary Figure S1: Boxplots of immunohistochemistry**


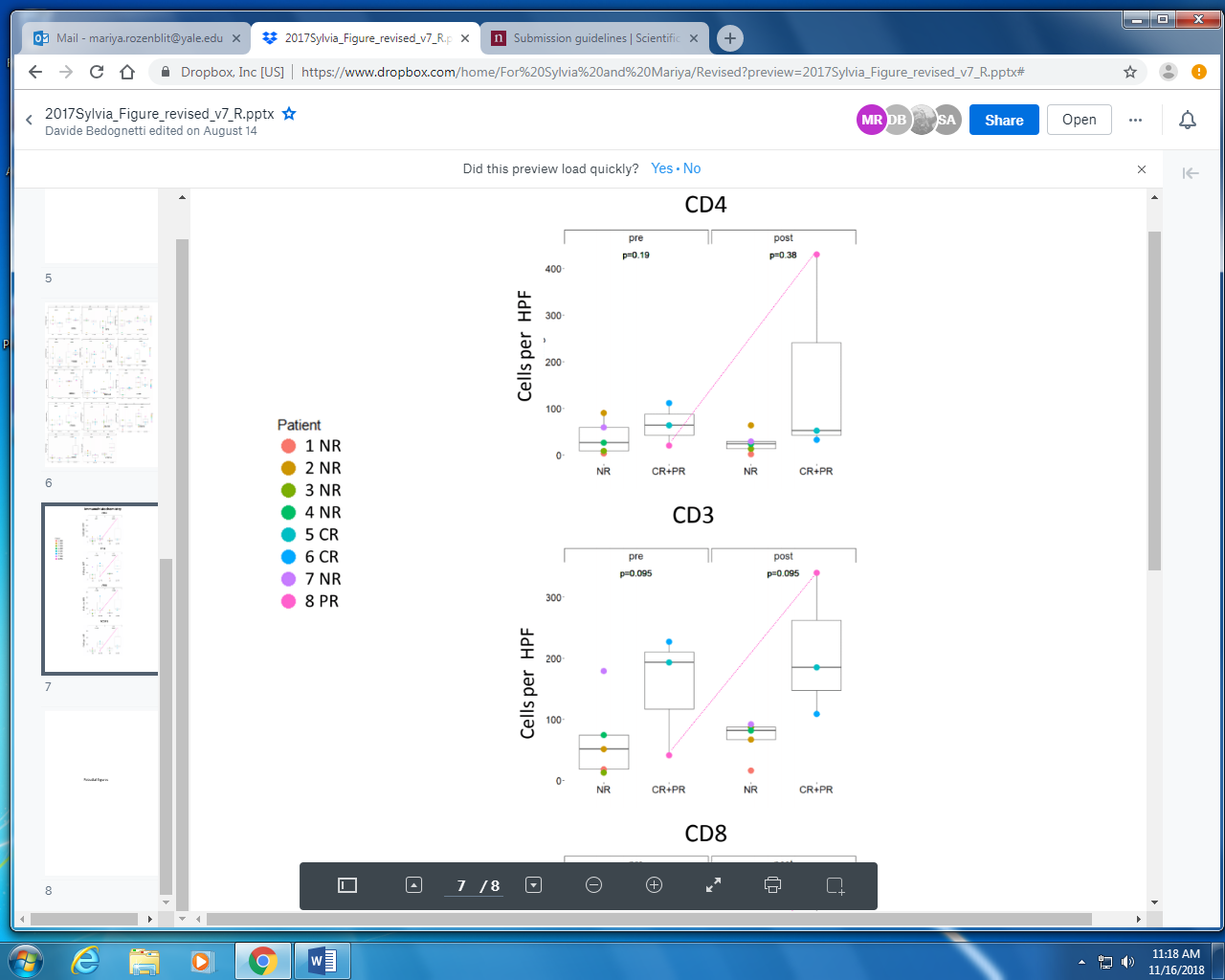


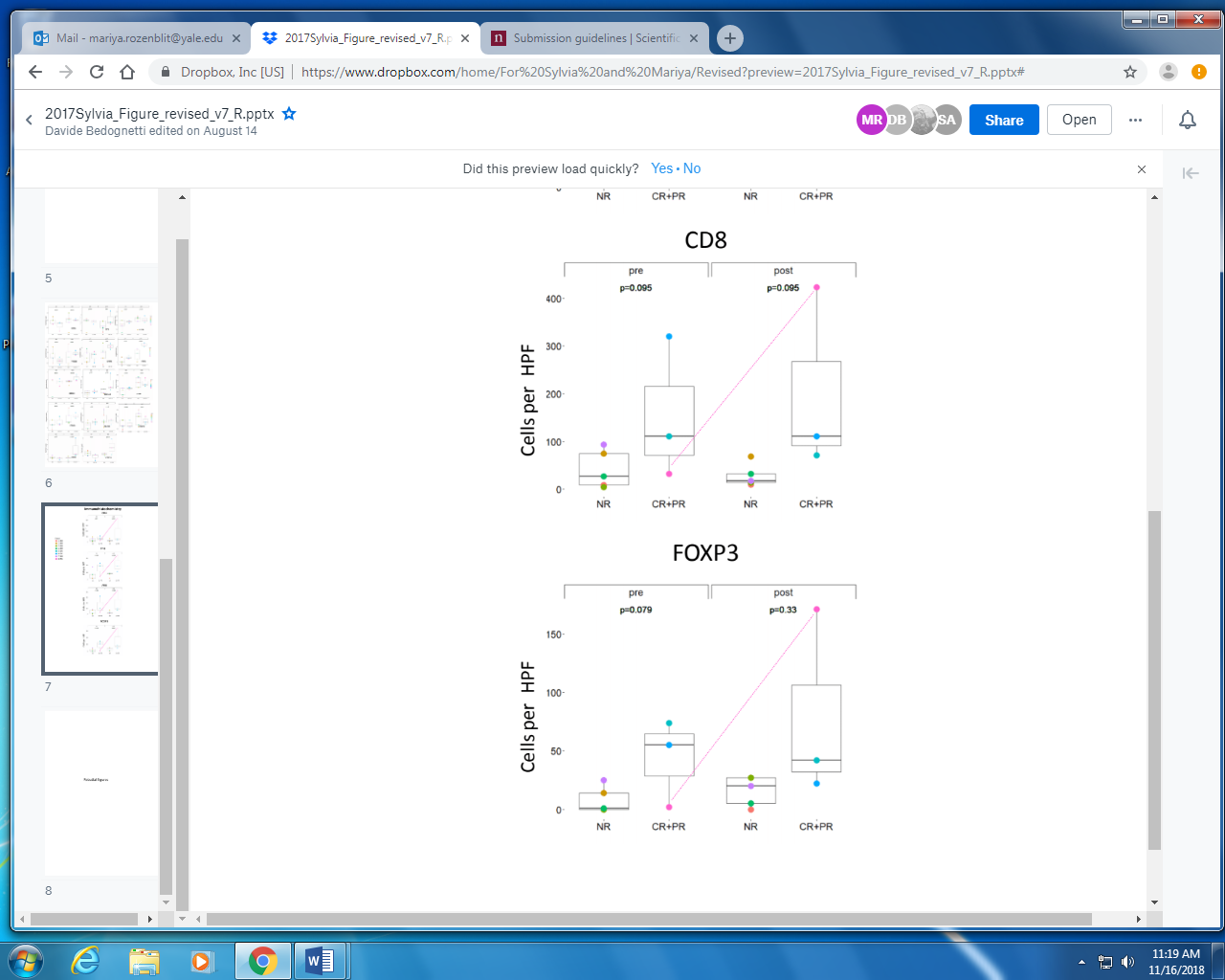


**Supplementary Figure 1. Boxplots of immunohistochemistry:** Cells per high power field for CD4, CD3, CD8, and FOXP3 immunohistochemistry staining are shown. *p* values displayed here were calculated using unpaired t-test including only CR and NR. PR data was displayed in the boxplots for comparison with the other two categories.

**Supplementary Figure S2: Boxplots of ICR genes**


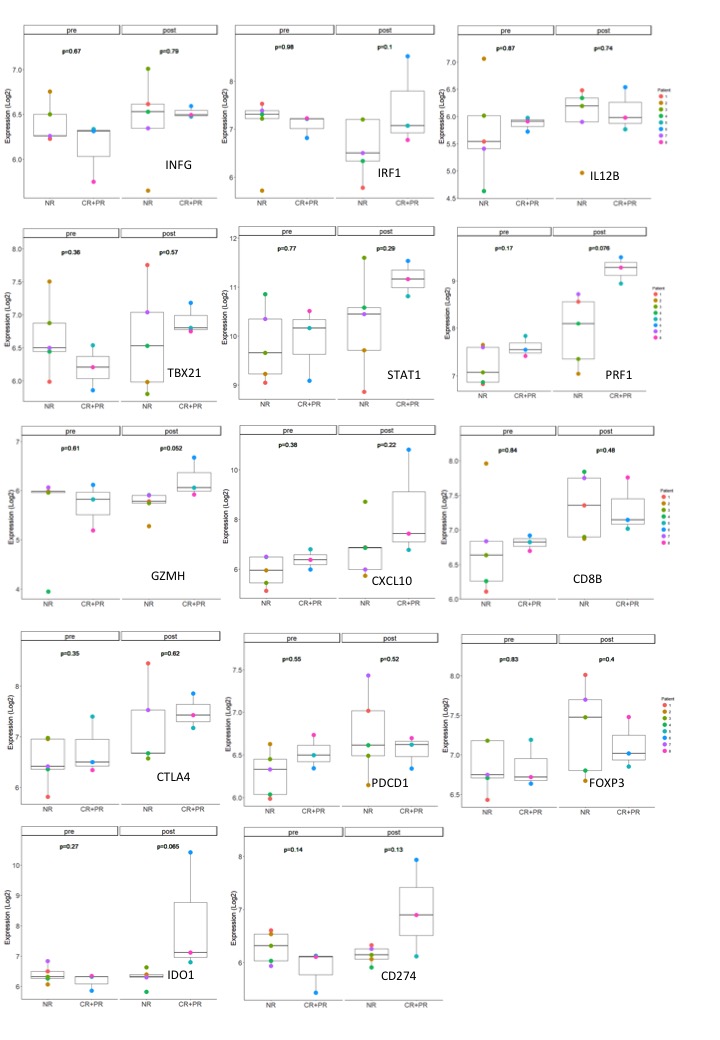


**Supplementary Figure S2.** Boxplots of ICR genes. *p* values displayed here were calculated using unpaired t-test including only CR and NR. PR data was displayed in the boxplots for comparison with the other two categories.

**Supplementary Table S1: Transcripts included in Nanostring panel**

| HUGO Name | NS Probe ID | Immune Response Category | Annotation |  |  |  |  |  |  |  |  |  |  |
| --- | --- | --- | --- | --- | --- | --- | --- | --- | --- | --- | --- | --- | --- |
| CCL1 | NM_002981.1:157 | Chemokines | Cell Type specific; Chemokines and receptors; Inflammatory response | | | | | |  |  |  |  |  |
| EBI3 | NM_005755.2:485 | Cytokines | Cell Type specific; Cytokines and receptors; Humoral immune response | | | | | |  |  |  |  |  |
| IDO1 | NM_002164.3:50 | Cytokines; T-Cell Functions | Cell Type specific; Cytokines and receptors; Chronic inflammatory response; Th2 orientation; T-cell proliferation | | | | | | | | |  |  |
| LAMP3 | NM_014398.3:1400 | Cell Functions | Basic cell functions; Cell Type specific; CD molecules | | | | |  |  |  |  |  |  |
| OAS3 | NM_006187.2:4980 | Cytokines; Pathogen Defense | Cell Type specific; Cytokines and receptors; Defense response to virus | | | | | |  |  |  |  |  |
| BLK | NM_001715.2:990 | B-Cell Functions | B-cell receptor signaling pathway; Cell Type specific | | | |  |  |  |  |  |  |  |
| CD19 | NM_001770.4:1770 | B-Cell Functions; Regulation | B-cell receptor signaling pathway; Cell Type specific; CD molecules; Regulation of immune response | | | | | | | |  |  |  |
| CR2 | NM_001006658.1:485 | B-cell Functions | B-cell activation; Cell Type specific; CD molecules; Innate immune response | | | | | |  |  |  |  |  |
| HLA-DOB | NM_002120.3:230 | Antigen Processing; Cytokines | Adaptive immune response; Antigen processing and presentation; Cell Type specific; Cytokines and receptors | | | | | | | | |  |  |
| MS4A1 | NM_152866.2:620 | B-Cell Functions | Basic cell functions; Cell Type specific; CD molecules; Humoral immune response | | | | | | |  |  |  |  |
| TNFRSF17 | NM_001192.2:635 | Cell Functions; TNF Superfamily | Basic cell functions; Cell Type specific; CD molecules; TNF superfamily members and their receptors | | | | | | | |  |  |  |
| CD8A | NM_001768.5:1320 | Antigen Processing; Pathogen Defense; T-Cell Functions | Adaptive immune response; Antigen processing and presentation; Cell Type specific; CD molecules; Defense response to virus; T-cell activation; T-cell differentiation | | | | | | | | | | |
| CD8B | NM_004931.3:440 | Regulation; T-Cell Functions | Adaptive immune response; Cell Type specific; CD molecules; Regulation of immune response; T-cell activation | | | | | | | | |  |  |
| FLT3LG | NM_001459.3:360 | Cytokines | Cell Type specific; Cytokines and receptors | | | |  |  |  |  |  |  |  |
| GZMM | NM_005317.2:669 | Cell Functions; Cytotoxicity | Adaptive immune response; Basic cell functions; Cell Type specific; Cytotoxicity; Innate immune response | | | | | | | |  |  |  |
| PRF1 | NM_005041.3:2120 | Cytotoxicity; Pathogen Defense | Adaptive immune response; Cell Type specific; Cytotoxicity; Defense response to tumor cell; Defense response to virus | | | | | | | | |  |  |
| GNLY | NM_006433.2:305 | Cytotoxicity; Pathogen Defense | Adaptive immune response; Cell Type specific; Cytotoxicity; Defense response to bacterium; Defense response to fungus | | | | | | | | | |  |
| GZMA | NM_006144.2:155 | Cell Functions; Cytotoxicity | Adaptive immune response; Basic cell functions; Cell Type specific; Cytotoxicity | | | | | | |  |  |  |  |
| GZMH | NM_033423.3:705 | Cell Functions; Cytotoxicity | Adaptive immune response; Basic cell functions; Cell Type specific; Cytotoxicity | | | | | | |  |  |  |  |
| KLRD1 | NM_002262.3:542 | NK Cell Functions; Regulation | Cell Type specific; NK cell functions; Regulation of immune response | | | | | |  |  |  |  |  |
| KLRF1 | NM_016523.1:275 | Cell Functions; NK Cell Functions | Basic cell functions; Cell Type specific; NK cell functions | | | | |  |  |  |  |  |  |
| CCL13 | NM_005408.2:320 | Chemokines | Cell Type specific; Chemokines and receptors; Inflammatory response | | | | | |  |  |  |  |  |
| CCL17 | NM_002987.2:229 | Chemokines | Cell Type specific; Chemokines and receptors; Inflammatory response | | | | | |  |  |  |  |  |
| CCL22 | NM_002990.3:797 | Chemokines; Pathogen Defense | Cell Type specific; Chemokines and receptors; Defense response to virus | | | | | |  |  |  |  |  |
| CD209 | NM_021155.2:1532 | Cell Functions | Basic cell functions; Cell Type specific; CD molecules | | | | |  |  |  |  |  |  |
| HSD11B1 | NM_181755.1:155 | Cell Functions | Basic cell functions; Cell Type specific | | |  |  |  |  |  |  |  |  |
| CCR3 | NM_001837.2:980 | Chemokines | Cell Type specific; CD molecules; Chemokines and receptors | | | | |  |  |  |  |  |  |
| IL5RA | NM_000564.3:210 | Cytokines | Cell Type specific; CD molecules; Cytokines and receptors | | | | |  |  |  |  |  |  |
| PTGDR2 | NM_004778.1:1835 | Cell Functions | Basic cell functions; Cell Type specific; CD molecules | | | | |  |  |  |  |  |  |
| SMPD3 | NM_018667.3:4733 | Cell Functions | Basic cell functions; Cell Type specific | | |  |  |  |  |  |  |  |  |
| THBS1 | NM_003246.2:3465 | Antigen Processing; Cell Cycle; Regulation | Cell cycle arrest; Cell Type specific; Chronic inflammatory response; Negative regulation of antigen processing | | | | | | | | |  |  |
| CD1A | NM_001763.2:1815 | Cell Functions | Basic cell functions; Cell Type specific; CD molecules | | | | |  |  |  |  |  |  |
| CD1B | NM_001764.2:1045 | Cell Functions | Basic cell functions; Cell Type specific; CD molecules | | | | |  |  |  |  |  |  |
| CD1E | NM_001042583.1:180 | Antigen Processing | Antigen processing and presentation; Cell Type specific; CD molecules | | | | | |  |  |  |  |  |
| F13A1 | NM_000129.3:3196 | Cell Functions | Basic cell functions; Cell Type specific | | |  |  |  |  |  |  |  |  |
| SYT17 | NM_016524.2:1150 | Cell Functions | Basic cell functions; Cell Type specific | | |  |  |  |  |  |  |  |  |
| APOE | NM_000041.2:96 | Transporter Functions | Cell Type specific; Lipid transporter activity | | | |  |  |  |  |  |  |  |
| CCL7 | NM_006273.2:120 | Chemokines | Cell Type specific; Chemokines and receptors; Inflammatory response | | | | | |  |  |  |  |  |
| CD68 | NM_001251.2:1140 | Cell Functions | Basic cell functions; Cell Type specific; CD molecules | | | | |  |  |  |  |  |  |
| CHIT1 | NM_003465.2:410 | Cell Functions | Basic cell functions; Cell Type specific | | |  |  |  |  |  |  |  |  |
| CXCL5 | NM_002994.3:250 | Chemokines | Cell Type specific; Chemokines and receptors | | | |  |  |  |  |  |  |  |
| MARCO | NM_006770.3:1434 | | Cell Type specific; Innate immune response | | | |  |  |  |  |  |  |  |
| MSR1 | NM_002445.3:326 | Cell Functions | Basic cell functions; Cell Type specific; CD molecules | | | | |  |  |  |  |  |  |
| CMA1 | NM_001836.2:561 | Regulation | Cell Type specific; Regulation of inflammatory response | | | | |  |  |  |  |  |  |
| CTSG | NM_001911.2:160 | Regulation; Pathogen Defense | Cell Type specific; Defense response to fungus; Positive regulation of immune response | | | | | | |  |  |  |  |
| KIT | NM_000222.2:2644 | Cell Functions | Basic cell functions; Cell Type specific; CD molecules | | | | |  |  |  |  |  |  |
| MS4A2 | NM_000139.3:661 | Chemokines | Cell Type specific; Humoral immune response; Inflammatory response | | | | | |  |  |  |  |  |
| PRG2 | NM_002728.4:256 | Pathogen Defense | Cell Type specific; Defense response to bacterium | | | |  |  |  |  |  |  |  |
| TPSAB1 | NM_003294.3:579 | Cell Functions | Basic cell functions; Cell Type specific | | |  |  |  |  |  |  |  |  |
| CSF3R | NM_156038.2:90 | Cytokines | Cell Type specific; CD molecules; Cytokines and receptors | | | | |  |  |  |  |  |  |
| FPR2 | NM_001462.3:1200 | | Cell Type specific; Inflammatory response | | | |  |  |  |  |  |  |  |
| MME | NM_000902.2:5059 | Cell Functions | Basic cell functions; Cell Type specific; CD molecules | | | | |  |  |  |  |  |  |
| FOXJ1 | NM_001454.3:815 | | Cell Type specific; Humoral immune response | | | |  |  |  |  |  |  |  |
| MPPED1 | NM_001044370.1:1486 | Cell Functions | Basic cell functions; Cell Type specific | | |  |  |  |  |  |  |  |  |
| PLA2G6 | NM_001004426.1:1954 | Cell Functions | Basic cell functions; Cell Type specific | | |  |  |  |  |  |  |  |  |
| RRAD | NM_004165.1:960 | Cell Functions | Basic cell functions; Cell Type specific | | |  |  |  |  |  |  |  |  |
| GTF3C1 | NM_001520.3:2904 | Cell Functions | Basic cell functions; Cell Type specific | | |  |  |  |  |  |  |  |  |
| GZMB | NM_004131.3:540 | Cell Functions; Cytotoxicity | Adaptive immune response; Basic cell functions; Cell Type specific; Cytotoxicity | | | | | | |  |  |  |  |
| IL21R | NM_021798.2:2080 | Cell Functions | Adaptive immune response; Basic cell functions; Cell Type specific; CD molecules | | | | | | |  |  |  |  |
| BCL2 | NM_000657.2:947 | Cell Cycle | Cell Type specific; G1/S transition of mitotic cell cycle | | | | |  |  |  |  |  |  |
| FUT5 | NM_002034.2:1726 | Cell Functions | Basic cell functions; Cell Type specific | | |  |  |  |  |  |  |  |  |
| NCR1 | NM_004829.5:602 | Cell Functions; NK Cell Functions | Basic cell functions; Cell Type specific; CD molecules; NK cell functions | | | | | |  |  |  |  |  |
| ZNF205 | NM_001031686.1:573 | Cell Functions | Basic cell functions; Cell Type specific | | |  |  |  |  |  |  |  |  |
| IL3RA | NM_002183.2:745 | Cell Functions | Basic cell functions; Cell Type specific; CD molecules | | | | |  |  |  |  |  |  |
| ANP32B | NM_006401.2:660 | Cell Functions | Basic cell functions; Cell Type specific | | |  |  |  |  |  |  |  |  |
| BATF | NM_006399.3:293 | Cell Functions | Basic cell functions; Cell Type specific | | |  |  |  |  |  |  |  |  |
| NUP107 | NM_020401.2:1002 | Cell Cycle | Cell Type specific; M phase of mitotic cell cycle | | | |  |  |  |  |  |  |  |
| CD28 | NM_001243078.1:2065 | | Cell Type specific; CD molecules; Humoral immune response; Inflammatory response to antigenic stimulus; T-cell activation; T-cell proliferation | | | | | | | | | | |
| ICOS | NM_012092.2:640 | Cell Functions | Adaptive immune response; Basic cell functions; Cell Type specific; CD molecules | | | | | | |  |  |  |  |
| CD2 | NM_001767.3:687 | Leukocyte Functions; T-Cell Functions | Cell Type specific; CD molecules; Leukocyte migration; T-cell activation; T-cell differentiation | | | | | | | |  |  |  |
| CD3E | NM_000733.2:75 | T-Cell Functions | Adaptive immune response; Cell Type specific; CD molecules; T-cell anergy; T-cell differentiation; T-cell proliferation | | | | | | | | |  |  |
| CD3G | NM_000073.2:404 | Regulation; T-Cell Functions | Adaptive immune response; Cell Type specific; CD molecules; Regulation of immune response; T-cell activation | | | | | | | | |  |  |
| CD6 | NM_006725.3:1280 | Cell Functions | Adaptive immune response; Basic cell functions; Cell Type specific; CD molecules | | | | | | |  |  |  |  |
| ATM | NM_000051.3:30 | Cell Cycle | Cell Type specific; DNA damage checkpoint; Induction of apoptosis | | | | | |  |  |  |  |  |
| DOCK9 | NM_001130048.1:1020 | Cell Functions | Basic cell functions; Cell Type specific | | |  |  |  |  |  |  |  |  |
| NEFL | NM_006158.3:3300 | Cell Functions | Basic cell functions; Cell Type specific | | |  |  |  |  |  |  |  |  |
| REPS1 | NM_001128617.2:1288 | Cell Functions | Basic cell functions; Cell Type specific | | |  |  |  |  |  |  |  |  |
| USP9Y | NM_004654.3:85 | Cell Functions | Basic cell functions; Cell Type specific | | |  |  |  |  |  |  |  |  |
| AKT3 | NM_181690.1:755 | Cell Functions | Basic cell functions; Cell Type specific | | |  |  |  |  |  |  |  |  |
| CCR2 | NM_001123041.2:743 | Cytokines | Cell Type specific; CD molecules; Cytokines and receptors; Inflammatory response | | | | | | |  |  |  |  |
| EWSR1 | NM_013986.3:452 | Cell Functions | Basic cell functions; Cell Type specific | | |  |  |  |  |  |  |  |  |
| LTK | NM_001135685.1:2418 | Cell Functions | Basic cell functions; Cell Type specific | | |  |  |  |  |  |  |  |  |
| NFATC4 | NM_001136022.2:2296 | | Cell Type specific; Inflammatory response | | | |  |  |  |  |  |  |  |
| BCL6 | NM_001706.2:675 | Regulation | Cell Type specific; Regulation of immune response | | | |  |  |  |  |  |  |  |
| CXCL13 | NM_006419.2:210 | Chemokines | Adaptive immune response; Cell Type specific; Chronic inflammatory response; Humoral immune response; Chemokines and receptors | | | | | | | | | | |
| MAF | NM_005360.4:888 | Cell Functions; T-Cell Functions | Basic cell functions; Cell Type specific; Th2 orientation | | | | |  |  |  |  |  |  |
| PDCD1 | NM_005018.1:175 | Regulation | Adaptive immune response; Cell Type specific; CD molecules; Humoral immune response; Negative regulation of immune response | | | | | | | | | |  |
| CD160 | NM_007053.2:500 | Regulation | Cell Type specific; CD molecules; Regulation of immune response | | | | |  |  |  |  |  |  |
| FEZ1 | NM_005103.4:426 | Cell Functions | Basic cell functions; Cell Type specific | | |  |  |  |  |  |  |  |  |
| TARP | NM_001003799.1:560 | Cell Functions | Basic cell functions; Cell Type specific | | |  |  |  |  |  |  |  |  |
| CD38 | NM_001775.2:460 | B-Cell Functions; Regulation | Adaptive immune response; Cell Type specific; CD molecules; Response to drug; Positive regulation of B-cell proliferation | | | | | | | | | |  |
| CSF2 | NM_000758.2:475 | Cytokines; Macrophage Functions; Regulation | Cell Type specific; Cytokines and receptors; Positive regulation of macrophages | | | | | |  |  |  |  |  |
| IFNG | NM_000619.2:970 | Cytokines; Interleukins; T-Cell Functions | Adaptive immune response; Cell Type specific; CD8-positive; Cytokines and receptors; Humoral immune response; Interleukins; Th1 orientation; T-cell differentiation | | | | | | | | | | |
| IL12RB2 | NM_001559.2:1315 | Cytokines; T-Cell Functions | Adaptive immune response; Cell Type specific; Cytokines and receptors; Th1 orientation | | | | | | |  |  |  |  |
| LTA | NM_000595.2:885 | Chemokines | Cell Type specific; Chronic inflammatory response to antigenic stimulus; Cytokines and receptors; Humoral immune response | | | | | | | | | |  |
| STAT4 | NM_003151.2:789 | Chemokines; Regulation; T-Cell Functions | Adaptive immune response; Cytokines and receptors; Cell Type specific; Transcription factors; Transcriptional regulators; Th1 orientation | | | | | | | | | | |
| TBX21 | NM_013351.1:890 | Regulation; T-Cell Functions | Adaptive immune response; Cell Type specific; Regulation of immune response; Th1 orientation | | | | | | | |  |  |  |
| CTLA4 | NM_005214.3:405 | B-Cell Functions; T-Cell Functions | Adaptive immune response; B-cell receptor signaling pathway; Cell Type specific; CD molecules; T-cell receptor signaling pathway | | | | | | | | | |  |
| IL17A | NM_002190.2:240 | Cytokines; Interleukins | Cell Type specific; Cytokines and receptors; Inflammatory response; Interleukins | | | | | | |  |  |  |  |
| IL17RA | NM_014339.6:482 | Cell Functions | Basic cell functions; Cell Type specific; CD molecules | | | | |  |  |  |  |  |  |
| RORC | NM_001001523.1:1350 | Cell Functions | Basic cell functions; Cell Type specific | | |  |  |  |  |  |  |  |  |
| CXCR6 | NM_006564.1:95 | Chemokines | Cell Type specific; CD molecules; Chemokines and receptors | | | | |  |  |  |  |  |  |
| GATA3 | NM_001002295.1:2835 | Cell Functions | Basic cell functions; Cell Type specific | | |  |  |  |  |  |  |  |  |
| IL26 | NM_018402.1:80 | Cytokines; Interleukins | Cell Type specific; Cytokines and receptors; Interleukins | | | | |  |  |  |  |  |  |
| LAIR2 | NM_002288.3:524 | Cell Functions | Basic cell functions; Cell Type specific; CD molecules | | | | |  |  |  |  |  |  |
| PMCH | NM_002674.2:172 | Cell Functions | Basic cell functions; Cell Type specific | | |  |  |  |  |  |  |  |  |
| SMAD2 | NM_005901.5:1678 | Cell Functions | Basic cell functions; Cell Type specific | | |  |  |  |  |  |  |  |  |
| STAT6 | NM_003153.3:2030 | Chemokines; Regulation; T-Cell Functions | Adaptive immune response; Cytokines and receptors; Cell Type specific; Th2 orientation; Transcription factors; Transcriptional regulators | | | | | | | | | | |
| FOXP3 | NM_014009.3:1230 | Cytokines; T-Cell Functions | Cell Type specific; Cytokines and receptors; T-cell mediated immunity; Chronic inflammatory response | | | | | | | |  |  |  |
| BAGE | NM_001187.1:399 | |  |  |  |  |  |  |  |  |  |  |  |
| CT45A1 | NM_001017417.1:866 | |  |  |  |  |  |  |  |  |  |  |  |
| CTAG1B | NM_001327.2:285 | |  |  |  |  |  |  |  |  |  |  |  |
| CTAGE1 | NM_172241.2:3365 | |  |  |  |  |  |  |  |  |  |  |  |
| CTCFL | NM_001269042.1:1100 | |  |  |  |  |  |  |  |  |  |  |  |
| DDX43 | NM_018665.2:1255 | |  |  |  |  |  |  |  |  |  |  |  |
| GAGE1 | NM_001040663.2:1642 | |  |  |  |  |  |  |  |  |  |  |  |
| MAGEA1 | NM_004988.4:476 | |  |  |  |  |  |  |  |  |  |  |  |
| MAGEA12 | NM_001166386.1:567 | |  |  |  |  |  |  |  |  |  |  |  |
| MAGEA3 | NM_005362.3:849 | |  |  |  |  |  |  |  |  |  |  |  |
| MAGEA4 | NM_001011548.1:778 | |  |  |  |  |  |  |  |  |  |  |  |
| MAGEB2 | NM_002364.4:1026 | |  |  |  |  |  |  |  |  |  |  |  |
| MAGEC1 | NM_005462.4:2920 | |  |  |  |  |  |  |  |  |  |  |  |
| MAGEC2 | NM_016249.3:860 | |  |  |  |  |  |  |  |  |  |  |  |
| PASD1 | NM_173493.2:820 | |  |  |  |  |  |  |  |  |  |  |  |
| PBK | NM_018492.2:1587 | |  |  |  |  |  |  |  |  |  |  |  |
| PRAME | NM_006115.3:1390 | |  |  |  |  |  |  |  |  |  |  |  |
| PRM1 | NM_002761.2:319 | |  |  |  |  |  |  |  |  |  |  |  |
| ROPN1 | NM_017578.2:195 | |  |  |  |  |  |  |  |  |  |  |  |
| SEMG1 | NM_003007.2:1290 | |  |  |  |  |  |  |  |  |  |  |  |
| SPA17 | NM_017425.3:175 | |  |  |  |  |  |  |  |  |  |  |  |
| SPACA3 | NM_173847.3:112 | |  |  |  |  |  |  |  |  |  |  |  |
| SPANXB1 | NM_032461.2:321 | |  |  |  |  |  |  |  |  |  |  |  |
| SPO11 | NM_198265.1:492 | |  |  |  |  |  |  |  |  |  |  |  |
| SSX1 | NM_005635.2:174 | |  |  |  |  |  |  |  |  |  |  |  |
| SSX4 | NM_005636.3:140 | |  |  |  |  |  |  |  |  |  |  |  |
| SYCP1 | NM_003176.2:230 | |  |  |  |  |  |  |  |  |  |  |  |
| TMEFF2 | NM_016192.2:972 | |  |  |  |  |  |  |  |  |  |  |  |
| TPTE | NM_199259.2:142 | |  |  |  |  |  |  |  |  |  |  |  |
| TTK | NM_003318.3:1200 | |  |  |  |  |  |  |  |  |  |  |  |
| ABCF1 | NM_001090.2:850 | |  |  |  |  |  |  |  |  |  |  |  |
| AGK | NM_018238.3:816 | |  |  |  |  |  |  |  |  |  |  |  |
| ALAS1 | NM_000688.4:1615 | |  |  |  |  |  |  |  |  |  |  |  |
| AMMECR1L | NM_001199140.1:3564 | |  |  |  |  |  |  |  |  |  |  |  |
| CC2D1B | NM_032449.2:4182 | |  |  |  |  |  |  |  |  |  |  |  |
| CNOT10 | NM_001256741.1:1962 | |  |  |  |  |  |  |  |  |  |  |  |
| CNOT4 | NM_001190848.1:795 | |  |  |  |  |  |  |  |  |  |  |  |
| COG7 | NM_153603.3:1492 | |  |  |  |  |  |  |  |  |  |  |  |
| DDX50 | NM_024045.1:1185 | |  |  |  |  |  |  |  |  |  |  |  |
| DHX16 | NM_001164239.1:2490 | |  |  |  |  |  |  |  |  |  |  |  |
| DNAJC14 | NM_032364.5:1166 | |  |  |  |  |  |  |  |  |  |  |  |
| EDC3 | NM_001142443.1:925 | |  |  |  |  |  |  |  |  |  |  |  |
| EIF2B4 | NM_172195.3:1390 | |  |  |  |  |  |  |  |  |  |  |  |
| ERCC3 | NM_000122.1:1950 | |  |  |  |  |  |  |  |  |  |  |  |
| FCF1 | NM_015962.4:1022 | |  |  |  |  |  |  |  |  |  |  |  |
| G6PD | NM_000402.2:1155 | |  |  |  |  |  |  |  |  |  |  |  |
| GPATCH3 | NM_022078.2:1685 | |  |  |  |  |  |  |  |  |  |  |  |
| GUSB | NM_000181.1:1350 | |  |  |  |  |  |  |  |  |  |  |  |
| HDAC3 | NM_003883.2:1455 | |  |  |  |  |  |  |  |  |  |  |  |
| HPRT1 | NM_000194.1:240 | |  |  |  |  |  |  |  |  |  |  |  |
| MRPS5 | NM_031902.3:390 | |  |  |  |  |  |  |  |  |  |  |  |
| MTMR14 | NM_022485.3:720 | |  |  |  |  |  |  |  |  |  |  |  |
| NOL7 | NM_016167.3:335 | |  |  |  |  |  |  |  |  |  |  |  |
| NUBP1 | NM_001278506.1:304 | |  |  |  |  |  |  |  |  |  |  |  |
| POLR2A | NM_000937.2:3775 | |  |  |  |  |  |  |  |  |  |  |  |
| PPIA | NM_021130.2:925 | |  |  |  |  |  |  |  |  |  |  |  |
| PRPF38A | NM_032864.3:335 | |  |  |  |  |  |  |  |  |  |  |  |
| SAP130 | NM_024545.3:3090 | |  |  |  |  |  |  |  |  |  |  |  |
| SDHA | NM_004168.1:230 | |  |  |  |  |  |  |  |  |  |  |  |
| SF3A3 | NM_006802.2:2060 | |  |  |  |  |  |  |  |  |  |  |  |
| TBP | NM_001172085.1:587 | |  |  |  |  |  |  |  |  |  |  |  |
| TLK2 | NM_006852.2:2335 | |  |  |  |  |  |  |  |  |  |  |  |
| TMUB2 | NM_024107.2:1485 | |  |  |  |  |  |  |  |  |  |  |  |
| TRIM39 | NM_021253.3:3140 | |  |  |  |  |  |  |  |  |  |  |  |
| TUBB | NM_178014.2:320 | |  |  |  |  |  |  |  |  |  |  |  |
| USP39 | NM_001256725.1:806 | |  |  |  |  |  |  |  |  |  |  |  |
| ZC3H14 | NM_001160103.1:2690 | |  |  |  |  |  |  |  |  |  |  |  |
| ZKSCAN5 | NM_014569.3:3688 | |  |  |  |  |  |  |  |  |  |  |  |
| ZNF143 | NM_003442.5:925 | |  |  |  |  |  |  |  |  |  |  |  |
| ZNF346 | NM_012279.2:2260 | |  |  |  |  |  |  |  |  |  |  |  |
| A2M | NM_000014.4:1685 | Chemokines | Chemokines and receptors; Innate immune response | | | | |  |  |  |  |  |  |
| ABCB1 | NM_000927.3:3910 | | CD molecules | |  |  |  |  |  |  |  |  |  |
| ABL1 | NM_005157.3:3200 | Cell Cycle; Regulation; Senescence | Regulation of cell cycle; S phase and DNA replication; Senescence initiators | | | | | |  |  |  |  |  |
| ADA | NM_000022.2:1300 | B-Cell Functions; T-Cell Functions | B-cell activation; B-cell differentiation; T-cell differentiation | | | | |  |  |  |  |  |  |
| AICDA | NM_020661.1:567 | B-Cell Functions | B-cell differentiation | |  |  |  |  |  |  |  |  |  |
| AIRE | NM_000383.2:1864 | | Humoral immune response | | |  |  |  |  |  |  |  |  |
| ALCAM | NM_001627.3:789 | Adhesion | Adaptive immune response; Adhesion; CD molecules | | | | |  |  |  |  |  |  |
| AMBP | NM_001633.3:597 | Regulation | Negative regulation of immune response | | | |  |  |  |  |  |  |  |
| AMICA1 | NM_153206.2:620 | Regulation | Regulation of immune response | | |  |  |  |  |  |  |  |  |
| ANXA1 | NM_000700.1:515 | Transporter Functions | Phagocytosis recognition and engulfment | | | |  |  |  |  |  |  |  |
| APP | NM_000484.3:1725 | | Innate immune response | |  |  |  |  |  |  |  |  |  |
| ARG1 | NM_000045.2:505 | | Response to drug | |  |  |  |  |  |  |  |  |  |
| ARG2 | NM_001172.3:1150 | | Response to drug | |  |  |  |  |  |  |  |  |  |
| ATF1 | NM_005171.2:710 | | Innate immune response | |  |  |  |  |  |  |  |  |  |
| ATF2 | NM_001256090.1:336 | | Innate immune response | |  |  |  |  |  |  |  |  |  |
| ATG10 | NM_001131028.1:985 | Transporter Functions | Genes responsible for protein transport | | | |  |  |  |  |  |  |  |
| ATG12 | NM_004707.2:25 | | Innate immune response | |  |  |  |  |  |  |  |  |  |
| ATG16L1 | NM_198890.2:1975 | Transporter Functions | Autophagic vacuole formation; Genes responsible for protein transport | | | | | |  |  |  |  |  |
| ATG5 | NM_004849.2:1104 | | Innate immune response | |  |  |  |  |  |  |  |  |  |
| ATG7 | NM_001136031.2:810 | Transporter Functions | Genes responsible for protein transport; Protein ubiquitination | | | | |  |  |  |  |  |  |
| AXL | NM_021913.2:2190 | | Innate immune response | |  |  |  |  |  |  |  |  |  |
| BAX | NM_138761.3:342 | Cell Cycle; Regulation | Co-Regulators of autophagy and apoptosis/cell cycle | | | | |  |  |  |  |  |  |
| BCL10 | NM_003921.2:1250 | | Adaptive immune response; Innate immune response | | | | |  |  |  |  |  |  |
| BCL2L1 | NM_001191.2:260 | | Innate immune response | |  |  |  |  |  |  |  |  |  |
| BID | NM_001196.2:1875 | Cell Cycle; Regulation | Co-Regulators of autophagy and apoptosis/cell cycle | | | | |  |  |  |  |  |  |
| BIRC5 | NM_001168.2:1215 | Cell Cycle | G2 phase and G2/M transition | | |  |  |  |  |  |  |  |  |
| BLNK | NM_013314.2:930 | | Humoral immune response | | |  |  |  |  |  |  |  |  |
| BMI1 | NM_005180.5:1145 | | Humoral immune response | | |  |  |  |  |  |  |  |  |
| BST1 | NM_004334.2:710 | | CD molecules; Humoral immune response | | | |  |  |  |  |  |  |  |
| BST2 | NM_004335.2:560 | | CD molecules; Humoral immune response; Innate immune response | | | | | |  |  |  |  |  |
| BTK | NM_000061.1:570 | | Adaptive immune response; Innate immune response | | | | |  |  |  |  |  |  |
| C1QA | NM_015991.2:718 | Complement | Complement pathway; Innate immune response | | | |  |  |  |  |  |  |  |
| C1QB | NM_000491.3:819 | Complement | Complement pathway; Innate immune response | | | |  |  |  |  |  |  |  |
| C1QBP | NM_001212.3:745 | Chemokines | CD molecules; Chemokines and receptors; Inflammatory response | | | | | |  |  |  |  |  |
| C1R | NM_001733.4:760 | Complement | Complement pathway; Innate immune response | | | |  |  |  |  |  |  |  |
| C1S | NM_001734.2:775 | Complement | Complement pathway; Innate immune response | | | |  |  |  |  |  |  |  |
| C2 | NM_000063.3:1075 | Complement | Innate immune response | |  |  |  |  |  |  |  |  |  |
| C3 | NM_000064.2:4396 | Regulation | Innate immune response; Regulation of immune response | | | | |  |  |  |  |  |  |
| C3AR1 | NM_004054.2:415 | Regulation | Regulation of inflammatory response | | |  |  |  |  |  |  |  |  |
| C4B | NM_001002029.3:4437 | Complement | Complement pathway; Innate immune response | | | |  |  |  |  |  |  |  |
| C4BPA | NM_000715.3:690 | Complement | Complement pathway; Innate immune response | | | |  |  |  |  |  |  |  |
| C5 | NM_001735.2:2592 | Complement | Complement pathway; Innate immune response | | | |  |  |  |  |  |  |  |
| C6 | NM_000065.2:3170 | Complement | Complement pathway; Innate immune response | | | |  |  |  |  |  |  |  |
| C7 | NM_000587.2:310 | Complement | Complement pathway; Innate immune response | | | |  |  |  |  |  |  |  |
| C8A | NM_000562.2:690 | Complement | Complement pathway; Innate immune response | | | |  |  |  |  |  |  |  |
| C8B | NM_000066.2:620 | Complement | Complement pathway; Innate immune response | | | |  |  |  |  |  |  |  |
| C8G | NM_000606.2:407 | Complement | Complement pathway; Innate immune response | | | |  |  |  |  |  |  |  |
| C9 | NM_001737.3:602 | Complement | Complement pathway; Innate immune response | | | |  |  |  |  |  |  |  |
| CAMP | NM_004345.3:220 | | Inflammatory response | |  |  |  |  |  |  |  |  |  |
| CARD11 | NM_032415.2:1075 | Regulation | Regulation of immune response | | |  |  |  |  |  |  |  |  |
| CARD9 | NM_052813.4:1525 | | Innate immune response | |  |  |  |  |  |  |  |  |  |
| CASP1 | NM_001223.3:971 | | Innate immune response | |  |  |  |  |  |  |  |  |  |
| CASP10 | NM_032977.3:20 | | Innate immune response | |  |  |  |  |  |  |  |  |  |
| CASP3 | NM_032991.2:685 | Cell Cycle; Regulation | Cell cycle checkpoint and cell cycle arrest; Co-Regulators of autophagy and apoptosis/cell cycle; Negative regulation of cell cycle | | | | | | | | | |  |
| CASP8 | NM_001228.4:301 | | Innate immune response | |  |  |  |  |  |  |  |  |  |
| CCL11 | NM_002986.2:378 | Chemokines | Chemokines and receptors; Chronic inflammatory response | | | | |  |  |  |  |  |  |
| CCL14 | NM_032963.3:274 | Chemokines | Chemokines and receptors | | |  |  |  |  |  |  |  |  |
| CCL15 | NM_032965.3:112 | Chemokines | Adaptive immune response; Chemokines and receptors; Inflammatory response | | | | | | |  |  |  |  |
| CCL16 | NM_004590.2:367 | Chemokines; Regulation | Chemokines and receptors; Regulation of inflammatory response; Humoral immune response | | | | | | | |  |  |  |
| CCL18 | NM_002988.2:585 | Chemokines | Chemokines and receptors; Anti-inflammatory cytokines | | | | |  |  |  |  |  |  |
| CCL19 | NM_006274.2:401 | Chemokines; Regulation | Chemokines and receptors; Anti-inflammatory cytokines; Regulation of inflammatory response | | | | | | | |  |  |  |
| CCL2 | NM_002982.3:123 | Chemokines | Chemokines and receptors; Humoral immune response | | | | |  |  |  |  |  |  |
| CCL20 | NM_004591.1:35 | Chemokines | Chemokines and receptors | | |  |  |  |  |  |  |  |  |
| CCL21 | NM_002989.2:180 | Chemokines; Regulation | Chemokines and receptors; Anti-inflammatory cytokines; Regulation of inflammatory response | | | | | | | |  |  |  |
| CCL23 | NM_145898.1:336 | Chemokines; Regulation | Chemokines and receptors; Regulation of inflammatory response | | | | |  |  |  |  |  |  |
| CCL24 | NM_002991.2:18 | Chemokines; Regulation | Chemokines and receptors; Regulation of inflammatory response | | | | |  |  |  |  |  |  |
| CCL25 | NM_005624.2:325 | Chemokines; Complement | Chemokines and receptors; Complement pathway; Inflammatory response | | | | | |  |  |  |  |  |
| CCL26 | NM_006072.4:184 | Chemokines | Chemokines and receptors; Inflammatory response | | | |  |  |  |  |  |  |  |
| CCL27 | NM_006664.2:304 | Chemokines | Chemokines and receptors | | |  |  |  |  |  |  |  |  |
| CCL28 | NM_148672.2:100 | Chemokines | Chemokines and receptors | | |  |  |  |  |  |  |  |  |
| CCL3 | NM_002983.2:159 | Chemokines; Regulation | Chemokines and receptors; Humoral immune response; Regulation of inflammatory response; T-cell polarization | | | | | | | | |  |  |
| CCL3L1 | NM_021006.4:421 | Cytokines | Cytokines and receptors | |  |  |  |  |  |  |  |  |  |
| CCL4 | NM_002984.2:35 | Chemokines; Regulation | Chemokines and receptors; Regulation of inflammatory response | | | | |  |  |  |  |  |  |
| CCL5 | NM_002985.2:280 | Chemokines; Cytokines | Cytokines and receptors; Chronic inflammatory response; Chemokines and receptors; Acute-phase response; Inflammatory response; Innate immune response | | | | | | | | | | |
| CCL8 | NM_005623.2:689 | Chemokines; Regulation | Chemokines and receptors; Regulation of inflammatory response | | | | |  |  |  |  |  |  |
| CCND3 | NM_001760.2:1215 | Cell Cycle | Regulation of cell cycle | |  |  |  |  |  |  |  |  |  |
| CCR1 | NM_001295.2:535 | Chemokines; Cytokines; Regulation; T-Cell Functions | CD molecules; Chemokines and receptors; Cytokines and receptors; Regulation of inflammatory response; T-cell polarization | | | | | | | | | |  |
| CCR4 | NM_005508.4:35 | Chemokines; Cytokines; Regulation; T-Cell Functions | CD molecules; Chemokines and receptors; Cytokines and receptors; Regulation of inflammatory response; T-cell polarization | | | | | | | | | |  |
| CCR5 | NM_000579.1:2730 | Cytokines; T-Cell Functions | CD molecules; Cytokines and receptors; T-cell polarization | | | | |  |  |  |  |  |  |
| CCR6 | NM_031409.2:935 | | CD molecules; Humoral immune response; Innate immune response | | | | | |  |  |  |  |  |
| CCR7 | NM_001838.2:1610 | Chemokines; Regulation | CD molecules; Chemokines and receptors; Humoral immune response; Regulation of inflammatory response | | | | | | | | |  |  |
| CCR9 | NM_031200.1:1095 | | CD molecules; Innate immune response | | | |  |  |  |  |  |  |  |
| CCRL2 | NM_003965.4:1110 | Chemokines | Chemokines and receptors; Inflammatory response | | | |  |  |  |  |  |  |  |
| CD14 | NM_000591.2:885 | | CD molecules; Innate immune response | | | |  |  |  |  |  |  |  |
| CD163 | NM_004244.4:1630 | Transporter Functions | CD molecules; Phagocytosis | | |  |  |  |  |  |  |  |  |
| CD164 | NM_006016.4:2575 | | CD molecules | |  |  |  |  |  |  |  |  |  |
| CD180 | NM_005582.2:1036 | | CD molecules; Innate immune response | | | |  |  |  |  |  |  |  |
| CD1C | NM_001765.2:750 | T-Cell Functions | CD molecules; T-cell activation | | |  |  |  |  |  |  |  |  |
| CD1D | NM_001766.3:1428 | T-Cell Functions | CD molecules; T-cell differentiation | | |  |  |  |  |  |  |  |  |
| CD200 | NM_005944.5:665 | Regulation | CD molecules; Regulation of immune response | | | |  |  |  |  |  |  |  |
| CD207 | NM_015717.2:995 | | CD molecules | |  |  |  |  |  |  |  |  |  |
| CD22 | NM_001771.2:2515 | | Adaptive immune response; CD molecules | | | |  |  |  |  |  |  |  |
| CD24 | NM_013230.2:95 | | CD molecules | |  |  |  |  |  |  |  |  |  |
| CD244 | NM_016382.2:1150 | | CD molecules | |  |  |  |  |  |  |  |  |  |
| CD247 | NM_198053.1:1490 | Regulation | CD molecules; Regulation of immune response | | | |  |  |  |  |  |  |  |
| CD274 | NM_014143.3:1245 | T-Cell Functions | CD molecules; T-cell polarization | | |  |  |  |  |  |  |  |  |
| CD276 | NM_001024736.1:2120 | Regulation | CD molecules; Regulation of immune response | | | |  |  |  |  |  |  |  |
| CD33 | NM_001177608.1:730 | | CD molecules | |  |  |  |  |  |  |  |  |  |
| CD34 | NM_001025109.1:1580 | Regulation | CD molecules; Regulation of immune response | | | |  |  |  |  |  |  |  |
| CD36 | NM_001001548.2:705 | Transporter Functions | CD molecules; Receptors involved in phagocytosis | | | |  |  |  |  |  |  |  |
| CD37 | NM_001774.2:535 | | Adaptive immune response; CD molecules | | | |  |  |  |  |  |  |  |
| CD3D | NM_000732.4:110 | Regulation | Adaptive immune response; CD molecules; Regulation of immune response | | | | | |  |  |  |  |  |
| CD3EAP | NM_012099.1:555 | Regulation | Adaptive immune response; CD molecules; Transcription factors | | | | |  |  |  |  |  |  |
| CD4 | NM_000616.4:975 | | Adaptive immune response; CD molecules; Innate immune response | | | | | |  |  |  |  |  |
| CD40 | NM_001250.4:1265 | Regulation | Adaptive immune response; CD molecules; Regulation of immune response | | | | | |  |  |  |  |  |
| CD44 | NM_001001392.1:429 | Senescence; Transporter Functions | Phagocytosis recognition and engulfment; Senescence pathway | | | | |  |  |  |  |  |  |
| CD46 | NM_172350.1:365 | | CD molecules; Innate immune response | | | |  |  |  |  |  |  |  |
| CD47 | NM_001777.3:897 | Regulation; T-Cell Functions; Transporter Functions | CD molecules; Phagocytosis recognition and engulfment; Regulators of T-cell activation | | | | | | |  |  |  |  |
| CD48 | NM_001778.2:270 | | CD molecules | |  |  |  |  |  |  |  |  |  |
| CD5 | NM_014207.2:1295 | T-Cell Functions; Regulation | Adaptive immune response; CD molecules; Regulators of Th1 and Th2 development | | | | | | |  |  |  |  |
| CD53 | NM_001040033.1:835 | | CD molecules; Adaptive immune response | | | |  |  |  |  |  |  |  |
| CD55 | NM_000574.3:101 | | CD molecules; Innate immune response | | | |  |  |  |  |  |  |  |
| CD58 | NM_001779.2:478 | | Adaptive immune response; CD molecules | | | |  |  |  |  |  |  |  |
| CD59 | NM_000611.4:730 | | CD molecules | |  |  |  |  |  |  |  |  |  |
| CD63 | NM_001780.4:350 | | CD molecules | |  |  |  |  |  |  |  |  |  |
| CD7 | NM_006137.6:440 | Regulation; T-Cell Functions | Adaptive immune response; CD molecules; Regulators of T-cell activation; Regulators of Th1 and Th2 development | | | | | | | | |  |  |
| CD70 | NM_001252.2:190 | Cytokines; TNF Superfamily; T-Cell Functions | Adaptive immune response; CD molecules; Cytokines and receptors; TNF superfamily members and their receptors | | | | | | | | |  |  |
| CD74 | NM_001025159.1:964 | | Adaptive immune response; CD molecules | | | |  |  |  |  |  |  |  |
| CD79A | NM_001783.3:695 | | Adaptive immune response; CD molecules | | | |  |  |  |  |  |  |  |
| CD79B | NM_021602.2:24 | B-Cell Functions | Adaptive immune response; B-cell activation; CD molecules | | | | |  |  |  |  |  |  |
| CD80 | NM_005191.3:1288 | B-Cell Functions; Regulation; T-Cell Functions | Adaptive immune response; B-cell activation; CD molecules; Regulators of T-cell activation; T-cell differentiation | | | | | | | | |  |  |
| CD81 | NM_004356.3:735 | Regulation | Adaptive immune response; CD molecules; Regulation of immune response | | | | | |  |  |  |  |  |
| CD83 | NM_004233.3:1960 | | CD molecules; Humoral immune response | | | |  |  |  |  |  |  |  |
| CD84 | NM_001184879.1:28 | | CD molecules | |  |  |  |  |  |  |  |  |  |
| CD86 | NM_175862.3:1265 | B-Cell Functions; Regulation; T-Cell Functions | Adaptive immune response; CD molecules; Defense response to virus; Regulators of T-cell activation; Th1 & Th2 differentiation; T-cell differentiation | | | | | | | | | | |
| CD9 | NM_001769.2:405 | | CD molecules | |  |  |  |  |  |  |  |  |  |
| CD96 | NM_005816.4:1355 | Regulation | CD molecules; Regulation of immune response | | | |  |  |  |  |  |  |  |
| CD97 | NM_078481.2:1370 | | CD molecules; Inflammatory response | | |  |  |  |  |  |  |  |  |
| CD99 | NM_002414.3:625 | | CD molecules | |  |  |  |  |  |  |  |  |  |
| CDH1 | NM_004360.2:535 | Regulation | CD molecules; Regulation of immune response | | | |  |  |  |  |  |  |  |
| CDH5 | NM_001795.3:3405 | | CD molecules | |  |  |  |  |  |  |  |  |  |
| CDK1 | NM_001786.4:178 | | Innate immune response | |  |  |  |  |  |  |  |  |  |
| CDKN1A | NM_000389.2:1975 | Cell Cycle; Regulation; Senescence | Cell cycle checkpoint and cell cycle arrest; Regulation of cell cycle; Senescence pathway; Senescence initiators interferon related | | | | | | | | | |  |
| CEACAM1 | NM_001712.3:2455 | Adhesion | Adhesion; CD molecules | |  |  |  |  |  |  |  |  |  |
| CEACAM6 | NM_002483.4:1217 | Adhesion | Adhesion; CD molecules | |  |  |  |  |  |  |  |  |  |
| CEACAM8 | NM_001816.3:825 | Adhesion; Chemokines | Adhesion; CD molecules; Chemokines and receptors | | | |  |  |  |  |  |  |  |
| CEBPB | NM_005194.2:1420 | | Acute-phase response | |  |  |  |  |  |  |  |  |  |
| CFB | NM_001710.5:2029 | | Innate immune response | |  |  |  |  |  |  |  |  |  |
| CFD | NM_001928.2:859 | | Innate immune response | |  |  |  |  |  |  |  |  |  |
| CFI | NM_000204.3:1780 | | Innate immune response | |  |  |  |  |  |  |  |  |  |
| CFP | NM_002621.2:380 | | Innate immune response | |  |  |  |  |  |  |  |  |  |
| CHUK | NM_001278.3:860 | | Innate immune response | |  |  |  |  |  |  |  |  |  |
| CKLF | NM_181640.2:275 | Chemokines | Chemokines and receptors; Inflammatory response | | | |  |  |  |  |  |  |  |
| CLEC4A | NM_194448.2:388 | | Innate immune response | |  |  |  |  |  |  |  |  |  |
| CLEC4C | NM_203503.1:570 | | CD molecules; Innate immune response | | | |  |  |  |  |  |  |  |
| CLEC5A | NM_013252.2:615 | | Innate immune response | |  |  |  |  |  |  |  |  |  |
| CLEC6A | NM_001007033.1:342 | | Innate immune response | |  |  |  |  |  |  |  |  |  |
| CLEC7A | NM_197954.2:55 | | Innate immune response | |  |  |  |  |  |  |  |  |  |
| CLU | NM_001831.2:2340 | | Innate immune response | |  |  |  |  |  |  |  |  |  |
| CMKLR1 | NM_004072.1:770 | Chemokines | Chemokines and receptors | | |  |  |  |  |  |  |  |  |
| COL3A1 | NM_000090.3:180 | Regulation | Negative regulation of immune response | | | |  |  |  |  |  |  |  |
| COLEC12 | NM_130386.2:900 | | Innate immune response | |  |  |  |  |  |  |  |  |  |
| CR1 | NM_000651.4:1695 | | CD molecules; Innate immune response | | | |  |  |  |  |  |  |  |
| CREB1 | NM_004379.3:4855 | | Innate immune response | |  |  |  |  |  |  |  |  |  |
| CREB5 | NM_182898.2:1885 | | Complement pathway; Inflammatory response | | | |  |  |  |  |  |  |  |
| CREBBP | NM_004380.2:8855 | | Innate immune response | |  |  |  |  |  |  |  |  |  |
| CRP | NM_000567.2:1521 | Transporter Functions | Acute-phase response; Humoral immune response; Inflammatory response; Innate immune response; Receptors involved in phagocytosis | | | | | | | | | | |
| CSF1 | NM_000757.4:823 | | Innate immune response | |  |  |  |  |  |  |  |  |  |
| CSF1R | NM_005211.2:3775 | | CD molecules; Innate immune response | | | |  |  |  |  |  |  |  |
| CSF2RB | NM_000395.2:3300 | Chemokines | CD molecules; Chemokines and receptors; Adaptive immune response | | | | | |  |  |  |  |  |
| CSF3 | NM_000759.3:851 | Cell Functions | Basic cell functions | |  |  |  |  |  |  |  |  |  |
| CTSH | NM_004390.3:344 | | Adaptive immune response | | |  |  |  |  |  |  |  |  |
| CTSL | NM_001912.4:1072 | | Adaptive immune response | | |  |  |  |  |  |  |  |  |
| CTSS | NM_004079.3:685 | | Adaptive immune response | | |  |  |  |  |  |  |  |  |
| CTSW | NM_001335.3:1075 | Transporter Functions | CD molecules; Phagocytosis | | |  |  |  |  |  |  |  |  |
| CX3CL1 | NM_002996.3:140 | Chemokines; T-Cell Functions | Adaptive immune response; Leukocyte activation; Chemokines and receptors; Th1 orientation | | | | | | | |  |  |  |
| CX3CR1 | NM_001337.3:1040 | Chemokines; Microglial Functions | Adaptive immune response; Chemokines and receptors; Microglial cell activation | | | | | | |  |  |  |  |
| CXCL1 | NM_001511.1:742 | Chemokines; Regulation | Chemokines and receptors; Regulation of inflammatory response | | | | |  |  |  |  |  |  |
| CXCL10 | NM_001565.1:40 | Chemokines; Cytokines; Pathogen Defense; Regulation; T-Cell Functions | Adaptive immune response; Chemokines and receptors; Cytokines and receptors; Defense response to virus; Innate immune response; Regulation of inflammatory response; Th1 orientation | | | | | | | | | | |
| CXCL11 | NM_005409.4:282 | Chemokines; T-Cell Functions | Adaptive immune response; Chemokines and receptors; Th1 orientation | | | | | |  |  |  |  |  |
| CXCL12 | NM_000609.5:210 | Chemokines | Chemokines and receptors | | |  |  |  |  |  |  |  |  |
| CXCL14 | NM_004887.4:1125 | Chemokines | Adaptive immune response; Chemokines and receptors; Inflammatory response | | | | | | |  |  |  |  |
| CXCL16 | NM_001100812.1:850 | Chemokines | Adaptive immune response; Chemokines and receptors | | | | |  |  |  |  |  |  |
| CXCL2 | NM_002089.3:854 | Chemokines; Regulation | Regulation of inflammatory response; Chemokines and receptors | | | | |  |  |  |  |  |  |
| CXCL3 | NM_002090.2:540 | Chemokines; Regulation | Chemokines and receptors; Regulation of inflammatory response | | | | |  |  |  |  |  |  |
| CXCL6 | NM_002993.3:539 | Chemokines; Regulation | Chemokines and receptors; Regulation of inflammatory response | | | | |  |  |  |  |  |  |
| CXCL8 | NM_000584.2:25 | Chemokines; Cytokines; Interleukins; Pathogen Defense; Regulation | Chemokines and receptors; Cytokines and receptors; Defense response to virus; Innate immune response; Interleukins; Regulation of inflammatory response | | | | | | | | | | |
| CXCL9 | NM_002416.1:1975 | Chemokines; Regulation; T-Cell Functions | Adaptive immune response; Chemokines and receptors; Regulation of inflammatory response; Th1 orientation | | | | | | | | |  |  |
| CXCR1 | NM_000634.2:1950 | Chemokines; Regulation | CD molecules; Chemokines and receptors; Regulation of inflammatory response | | | | | | |  |  |  |  |
| CXCR2 | NM_001557.2:2055 | Chemokines; Regulation | CD molecules; Chemokines and receptors; Regulation of inflammatory response | | | | | | |  |  |  |  |
| CXCR3 | NM_001504.1:80 | Chemokines; T-Cell Functions | Adaptive immune response; CD molecules; Chemokines and receptors; T-cell polarization | | | | | | |  |  |  |  |
| CXCR4 | NM_003467.2:1335 | Cell Cycle; Chemokines; Regulation; T-Cell Functions | Adaptive immune response; CD molecules; Chemokines and receptors; Co-Regulators of autophagy and apoptosis/cell cycle; T-cell polarization | | | | | | | | | | |
| CXCR5 | NM_001716.3:2618 | Chemokines; B-Cell Functions | Adaptive immune response; B-cell activation; CD molecules; Chemokines and receptors | | | | | | |  |  |  |  |
| CYBB | NM_000397.3:2686 | | Innate immune response | |  |  |  |  |  |  |  |  |  |
| CYFIP2 | NM_001037332.2:4043 | | Innate immune response | |  |  |  |  |  |  |  |  |  |
| CYLD | NM_015247.1:2890 | | Innate immune response | |  |  |  |  |  |  |  |  |  |
| DDX58 | NM_014314.3:2130 | | Innate immune response | |  |  |  |  |  |  |  |  |  |
| DEFB1 | NM_005218.3:40 | | Innate immune response | |  |  |  |  |  |  |  |  |  |
| DMBT1 | NM_007329.2:3712 | | Innate immune response | |  |  |  |  |  |  |  |  |  |
| DPP4 | NM_001935.3:2700 | Regulation; T-Cell Functions | CD molecules; Regulators of T-cell activation | | | |  |  |  |  |  |  |  |
| DUSP4 | NM_057158.2:3115 | | Innate immune response | |  |  |  |  |  |  |  |  |  |
| DUSP6 | NM_001946.2:1535 | | Innate immune response | |  |  |  |  |  |  |  |  |  |
| ECSIT | NM_001142464.2:1318 | | Innate immune response | |  |  |  |  |  |  |  |  |  |
| EGR1 | NM_001964.2:1505 | Senescence; T-Cell Functions | T-cell differentiation; Senescence initiators interferon related | | | | |  |  |  |  |  |  |
| EGR2 | NM_000399.3:1891 | Regulation | Transcriptional regulators | | |  |  |  |  |  |  |  |  |
| ELANE | NM_001972.2:195 | Regulation | Positive regulation of immune response | | | |  |  |  |  |  |  |  |
| ELK1 | NM_005229.3:2350 | | Innate immune response | |  |  |  |  |  |  |  |  |  |
| ENG | NM_001114753.1:1480 | | CD molecules | |  |  |  |  |  |  |  |  |  |
| ENTPD1 | NM_001098175.1:8830 | | Adaptive immune response; CD molecules | | | |  |  |  |  |  |  |  |
| EOMES | NM_005442.2:1670 | T-Cell Functions | CD8-positive; T-cell differentiation | | |  |  |  |  |  |  |  |  |
| EP300 | NM_001429.2:715 | | Innate immune response | |  |  |  |  |  |  |  |  |  |
| EPCAM | NM_002354.1:415 | Adhesion; Cell Functions | Adhesion; Basic cell functions; CD molecules | | | |  |  |  |  |  |  |  |
| ETS1 | NM_005238.3:4625 | Senescence | Senescence pathway | |  |  |  |  |  |  |  |  |  |
| F12 | NM_000505.3:1862 | | Innate immune response | |  |  |  |  |  |  |  |  |  |
| F2RL1 | NM_005242.3:940 | T-Cell Functions | T-cell activation | |  |  |  |  |  |  |  |  |  |
| FADD | NM_003824.2:1560 | | Innate immune response | |  |  |  |  |  |  |  |  |  |
| FAS | NM_000043.3:90 | B-Cell Functions; Regulation; TNF Superfamily; Transporter Functions; T-Cell Functions | B-cell activation; CD molecules; Co-Regulators of autophagy and apoptosis/cell cycle; Receptors involved in phagocytosis; T-cell regulators; TNF superfamily members and their receptors | | | | | | | | | | |
| FCER1A | NM_002001.2:114 | | Inflammatory response | |  |  |  |  |  |  |  |  |  |
| FCER1G | NM_004106.1:36 | Regulation | Positive regulation of immune response | | | |  |  |  |  |  |  |  |
| FCER2 | NM_002002.4:420 | | Adaptive immune response; CD molecules; Inflammatory response | | | | | |  |  |  |  |  |
| FCGR1A | NM_000566.3:1545 | | CD molecules; Innate immune response | | | |  |  |  |  |  |  |  |
| FCGR2A | NM_021642.3:60 | Transporter Functions | CD molecules; Receptors involved in phagocytosis | | | |  |  |  |  |  |  |  |
| FCGR2B | NM_001002273.1:870 | Regulation | CD molecules; Regulation of immune response | | | |  |  |  |  |  |  |  |
| FCGR3A | NM_000569.6:1644 | Regulation | CD molecules; Regulation of immune response | | | |  |  |  |  |  |  |  |
| FLT3 | NM_004119.1:580 | | CD molecules | |  |  |  |  |  |  |  |  |  |
| FN1 | NM_212482.1:1776 | Senescence | Senescence pathway | |  |  |  |  |  |  |  |  |  |
| FOS | NM_005252.2:1475 | | Innate immune response | |  |  |  |  |  |  |  |  |  |
| FUT7 | NM_004479.3:1710 | Leukocyte Functions | Leukocyte migration | |  |  |  |  |  |  |  |  |  |
| FYN | NM_002037.3:765 | Transporter Functions | Phagyocytosis signal transduction | | |  |  |  |  |  |  |  |  |
| GPI | NM_000175.2:1695 | | Humoral immune response | | |  |  |  |  |  |  |  |  |
| GZMK | NM_002104.2:700 | Cell Functions; Cytotoxicity | Adaptive immune response; Basic cell functions; Cytotoxicity; Innate immune response | | | | | | |  |  |  |  |
| HAMP | NM_021175.2:99 | | Innate immune response | |  |  |  |  |  |  |  |  |  |
| HCK | NM_002110.2:260 | Leukocyte Functions | Innate immune response; Leukocyte migration | | | |  |  |  |  |  |  |  |
| HLA-A | NM_002116.5:1000 | Antigen Processing; Cytotoxicity; Regulation | Adaptive immune response; Antigen processing and presentation; Cytotoxicity; Regulation of immune response | | | | | | | | |  |  |
| HLA-B | NM_005514.6:937 | Antigen Processing; Cytotoxicity; Regulation | Adaptive immune response; Antigen processing and presentation; Cytotoxicity; Regulation of immune response | | | | | | | | |  |  |
| HLA-C | NM_002117.4:895 | Antigen Processing; Cytotoxicity; Regulation | Adaptive immune response; Antigen processing and presentation; Cytotoxicity; Regulation of immune response | | | | | | | | |  |  |
| HLA-DMA | NM_006120.3:380 | Antigen Processing; Regulation | Adaptive immune response; Antigen processing and presentation; Positive regulation of immune response | | | | | | | | |  |  |
| HLA-DMB | NM_002118.3:20 | Antigen Processing | Adaptive immune response; Antigen processing and presentation | | | | |  |  |  |  |  |  |
| HLA-DPA1 | NM_033554.2:857 | Antigen Processing | Adaptive immune response; Antigen processing and presentation | | | | |  |  |  |  |  |  |
| HLA-DPB1 | NM_002121.4:931 | Antigen Processing | Adaptive immune response; Antigen processing and presentation | | | | |  |  |  |  |  |  |
| HLA-DQA1 | NM_002122.3:261 | Antigen Processing | Adaptive immune response; Antigen processing and presentation | | | | |  |  |  |  |  |  |
| HLA-DQB1 | NM_002123.3:384 | Antigen Processing | Adaptive immune response; Antigen processing and presentation | | | | |  |  |  |  |  |  |
| HLA-DRA | NM_019111.3:335 | Antigen Processing | Adaptive immune response; Antigen processing and presentation | | | | |  |  |  |  |  |  |
| HLA-DRB3 | NM_022555.3:698 | Antigen Processing | Adaptive immune response; Antigen processing and presentation | | | | |  |  |  |  |  |  |
| HLA-DRB4 | NM_021983.4:194 | Antigen Processing | Adaptive immune response; Antigen processing and presentation | | | | |  |  |  |  |  |  |
| HLA-E | NM_005516.4:1204 | Regulation | Regulation of immune response | | |  |  |  |  |  |  |  |  |
| HLA-G | NM_002127.4:1180 | Regulation | Regulation of immune response | | |  |  |  |  |  |  |  |  |
| HMGB1 | NM_002128.4:208 | Regulation | Inflammatory response to antigenic stimulus; Innate immune response | | | | | |  |  |  |  |  |
| HRAS | NM_005343.2:396 | Senescence | Senescence pathway | |  |  |  |  |  |  |  |  |  |
| ICAM1 | NM_000201.2:2253 | Adhesion; Regulation | Adhesion; CD molecules; Regulation of immune response | | | | |  |  |  |  |  |  |
| ICAM2 | NM_000873.3:415 | Adhesion; Regulation | Adhesion; CD molecules; Regulation of immune response | | | | |  |  |  |  |  |  |
| ICAM3 | NM_002162.3:1225 | Adhesion; Regulation | Adhesion; CD molecules; Regulation of immune response | | | | |  |  |  |  |  |  |
| ICAM4 | NM_001039132.1:463 | Adhesion; Regulation | Adhesion; CD molecules; Regulation of immune response | | | | |  |  |  |  |  |  |
| ICOSLG | NM_015259.4:1190 | B-Cell Functions; Regulation; T-Cell Functions | Adaptive immune response; B-cell activation; CD molecules; Regulators of T-cell activation; T-cell proliferation | | | | | | | | |  |  |
| IFI16 | NM_005531.1:2255 | Chemokines | Chemokines and receptors | | |  |  |  |  |  |  |  |  |
| IFI27 | NM_005532.3:390 | Chemokines | Chemokines and receptors | | |  |  |  |  |  |  |  |  |
| IFI35 | NM_005533.3:415 | Chemokines | Chemokines and receptors | | |  |  |  |  |  |  |  |  |
| IFIH1 | NM_022168.2:185 | | Innate immune response | |  |  |  |  |  |  |  |  |  |
| IFIT1 | NM_001548.3:1440 | Chemokines | Chemokines and receptors | | |  |  |  |  |  |  |  |  |
| IFIT2 | NM_001547.4:1995 | Chemokines | Chemokines and receptors | | |  |  |  |  |  |  |  |  |
| IFITM1 | NM_003641.3:482 | Regulation | CD molecules; Innate immune response; Regulation of immune response | | | | | |  |  |  |  |  |
| IFITM2 | NM_006435.2:390 | | Innate immune response | |  |  |  |  |  |  |  |  |  |
| IFNA1 | NM_024013.1:585 | Interleukins | Innate immune response; Interleukins | | |  |  |  |  |  |  |  |  |
| IFNA17 | NM_021268.2:291 | Interleukins | Innate immune response; Interleukins | | |  |  |  |  |  |  |  |  |
| IFNA2 | NM_000605.3:611 | Interleukins | Innate immune response; Interleukins | | |  |  |  |  |  |  |  |  |
| IFNA7 | NM_021057.2:215 | Interleukins | Innate immune response; Interleukins | | |  |  |  |  |  |  |  |  |
| IFNA8 | NM_002170.3:604 | Interleukins | Innate immune response; Interleukins | | |  |  |  |  |  |  |  |  |
| IFNAR1 | NM_000629.2:3123 | Pathogen Defense | Defense response to virus | | |  |  |  |  |  |  |  |  |
| IFNAR2 | NM_000874.3:631 | Chemokines | Chemokines and receptors | | |  |  |  |  |  |  |  |  |
| IFNB1 | NM_002176.2:610 | | Adaptive immune response; Humoral immune response; Innate immune response | | | | | | |  |  |  |  |
| IFNGR1 | NM_000416.1:1140 | T-Cell Functions | Adaptive immune response; CD molecules; Innate immune response; Th1 orientation | | | | | | |  |  |  |  |
| IFNL1 | NM_172140.1:233 | Cytokines; Interleukins | Cytokines and receptors; Interleukins | | |  |  |  |  |  |  |  |  |
| IFNL2 | NM_172138.1:589 | Chemokines | Chemokines and receptors | | |  |  |  |  |  |  |  |  |
| IGF1R | NM_000875.2:455 | Senescence | CD molecules; Senescence initiators | | |  |  |  |  |  |  |  |  |
| IGF2R | NM_000876.1:2605 | | CD molecules | |  |  |  |  |  |  |  |  |  |
| IGLL1 | NM_020070.2:188 | | CD molecules | |  |  |  |  |  |  |  |  |  |
| IKBKB | NM_001556.1:1995 | | Innate immune response | |  |  |  |  |  |  |  |  |  |
| IKBKE | NM_014002.2:2470 | | Innate immune response | |  |  |  |  |  |  |  |  |  |
| IKBKG | NM_003639.2:470 | | Innate immune response | |  |  |  |  |  |  |  |  |  |
| IL10 | NM_000572.2:230 | Interleukins | Immunosuppression; Interleukins | | |  |  |  |  |  |  |  |  |
| IL10RA | NM_001558.2:150 | Cytokines | CD molecules; Cytokines and receptors | | |  |  |  |  |  |  |  |  |
| IL11 | NM_000641.2:1145 | B-Cell Functions; Cytokines; Interleukins | Anti-inflammatory cytokines; B-cell differentiation; Interleukins | | | | |  |  |  |  |  |  |
| IL11RA | NM_147162.1:400 | Chemokines | Chemokines and receptors | | |  |  |  |  |  |  |  |  |
| IL12A | NM_000882.2:775 | Cytokines; Interleukins; NK Cell Functions; Regulation; T-Cell Functions | Adaptive immune response; Anti-inflammatory cytokines; Interleukins; NK cell activation; Regulators of Th1 and Th2 development; Th1 orientation; Th1 & Th2 differentiation; T-cell polarization | | | | | | | | | | |
| IL12B | NM_002187.2:1435 | Cytokines; Interleukins; NK Cell Functions; T-Cell Functions | Adaptive immune response; Anti-inflammatory cytokines; Cytokines and receptors; Interleukins; NK cell activation; Th1 orientation; Th1 & Th2 differentiation; T-cell differentiation; T-cell proliferation | | | | | | | | | | |
| IL12RB1 | NM_005535.1:225 | T-Cell Functions | Adaptive immune response; CD molecules; T-cell polarization; Th1 & Th2 differentiation; Th1 orientation | | | | | | | |  |  |  |
| IL13 | NM_002188.2:516 | Cytokines; Interleukins; T-Cell Functions | Anti-inflammatory cytokines; Cytokines and receptors; Interleukins; Macrophage activation; Regulators of Th1 and Th2 development; Th2 orientation | | | | | | | | | | |
| IL13RA1 | NM_001560.2:1230 | Cytokines; T-Cell Functions | CD molecules; Cytokines and receptors; Th2 orientation | | | | |  |  |  |  |  |  |
| IL13RA2 | NM_000640.2:400 | Chemokines; T-Cell Functions | CD molecules; Chemokines and receptors; Th2 orientation | | | | |  |  |  |  |  |  |
| IL15 | NM_172174.1:1685 | Interleukins; Regulation | Adaptive immune response; Interleukins; Positive regulation of immune response | | | | | | |  |  |  |  |
| IL15RA | NM_002189.2:505 | Chemokines | Adaptive immune response; CD molecules; Chemokines and receptors | | | | | |  |  |  |  |  |
| IL16 | NM_004513.4:1262 | Interleukins | Interleukins |  |  |  |  |  |  |  |  |  |  |
| IL17B | NM_014443.2:177 | Chemokines; Interleukins | Chemokines and receptors; Interleukins | | | |  |  |  |  |  |  |  |
| IL17F | NM_052872.3:210 | Interleukins | Interleukins |  |  |  |  |  |  |  |  |  |  |
| IL17RB | NM_018725.3:225 | Chemokines | Chemokines and receptors | | |  |  |  |  |  |  |  |  |
| IL18 | NM_001562.2:48 | Interleukins; T-Cell Functions | Interleukins; Th1 orientation | | |  |  |  |  |  |  |  |  |
| IL18R1 | NM_003855.2:2025 | T-Cell Functions | CD molecules; Innate immune response; Th1 orientation | | | | |  |  |  |  |  |  |
| IL18RAP | NM_003853.2:2412 | T-Cell Functions | CD molecules; Innate immune response; Th1 orientation | | | | |  |  |  |  |  |  |
| IL19 | NM_013371.3:1030 | Chemokines; Interleukins | Chemokines and receptors; Interleukins | | | |  |  |  |  |  |  |  |
| IL1A | NM_000575.3:1085 | Cytokines; Interleukins | Acute-phase response; Cytokines and receptors; Inflammatory response; Innate immune response; Interleukins | | | | | | | | |  |  |
| IL1B | NM_000576.2:840 | Chemokines; Cytokines; Interleukins; Pathogen Defense; Regulation | Innate immune response; Chemokines and receptors; Cytokines and receptors; Defense response to virus; Interleukins; Regulation of inflammatory response | | | | | | | | | | |
| IL1R1 | NM_000877.2:4295 | | CD molecules; Innate immune response | | | |  |  |  |  |  |  |  |
| IL1R2 | NM_173343.1:113 | Cytokines | CD molecules; Cytokines and receptors | | |  |  |  |  |  |  |  |  |
| IL1RAP | NM_002182.2:460 | | Innate immune response | |  |  |  |  |  |  |  |  |  |
| IL1RAPL2 | NM_017416.1:1800 | | Innate immune response | |  |  |  |  |  |  |  |  |  |
| IL1RL1 | NM_016232.4:700 | | Innate immune response | |  |  |  |  |  |  |  |  |  |
| IL1RL2 | NM_003854.2:595 | | Innate immune response | |  |  |  |  |  |  |  |  |  |
| IL1RN | NM_000577.3:480 | Cytokines; Interleukins | Acute-phase response; Cytokines and receptors; Interleukins | | | | |  |  |  |  |  |  |
| IL2 | NM_000586.2:300 | Cytokines; T-Cell Functions; Regulation | Adaptive immune response; Anti-inflammatory cytokines; B-cell activation; Cytokines and receptors; Innate immune response; Interleukins; Th1 & Th2 differentiation; T-cell differentiation; T-cell polarization; T-cell regulators | | | | | | | | | | |
| IL21 | NM_021803.2:65 | Cytokines; Interleukins | Adaptive immune response; Cytokines and receptors; Interleukins | | | | |  |  |  |  |  |  |
| IL22 | NM_020525.4:319 | Cytokines | Acute-phase response; Anti-inflammatory cytokines; Interleukins | | | | |  |  |  |  |  |  |
| IL22RA1 | NM_021258.2:2524 | Chemokines | Chemokines and receptors | | |  |  |  |  |  |  |  |  |
| IL22RA2 | NM_181310.1:290 | Chemokines | Chemokines and receptors | | |  |  |  |  |  |  |  |  |
| IL23A | NM_016584.2:411 | Interleukins | Innate immune response; Interleukins | | |  |  |  |  |  |  |  |  |
| IL23R | NM_144701.2:710 | Cytokines | Cytokines and receptors | |  |  |  |  |  |  |  |  |  |
| IL24 | NM_181339.1:1016 | Cytokines; Interleukins | Anti-inflammatory cytokines; Interleukins | | | |  |  |  |  |  |  |  |
| IL25 | NM_022789.2:1027 | Interleukins | Inflammatory response to antigenic stimulus; Interleukins | | | | |  |  |  |  |  |  |
| IL27 | NM_145659.3:143 | Interleukins | Innate immune response; Interleukins | | |  |  |  |  |  |  |  |  |
| IL2RA | NM_000417.1:1000 | Regulation | Adaptive immune response; CD molecules; Inflammatory response to antigenic stimulus; Negative regulation of immune response | | | | | | | | | |  |
| IL2RB | NM_000878.2:1980 | Cytokines | Adaptive immune response; CD molecules; Cytokines and receptors | | | | | |  |  |  |  |  |
| IL2RG | NM_000206.1:595 | Chemokines | Adaptive immune response; CD molecules; Chemokines and receptors | | | | | |  |  |  |  |  |
| IL3 | NM_000588.3:130 | Regulation; T-Cell Functions | Interleukins; Regulators of Th1 and Th2 development | | | | |  |  |  |  |  |  |
| IL32 | NM_004221.4:358 | Chemokines; Interleukins | Chemokines and receptors; Interleukins | | | |  |  |  |  |  |  |  |
| IL34 | NM_152456.1:860 | Interleukins | Innate immune response; Interleukins | | |  |  |  |  |  |  |  |  |
| IL4 | NM_000589.2:625 | Interleukins; Regulation; T-Cell Functions | Interleukins; Regulation of immune response; Th2 orientation | | | | |  |  |  |  |  |  |
| IL4R | NM_000418.2:705 | Cytokines; T-Cell Functions | CD molecules; Cytokines and receptors; Th1 & Th2 differentiation; Th2 orientation; T-cell polarization | | | | | | | |  |  |  |
| IL5 | NM_000879.2:105 | Cytokines; Interleukins; Regulation; T-Cell Functions | Cytokines and receptors; Interleukins; Regulators of Th1 and Th2 development; Th2 orientation; T-cell polarization | | | | | | | | |  |  |
| IL6 | NM_000600.1:220 | Interleukins | Humoral immune response; Interleukins | | | |  |  |  |  |  |  |  |
| IL6R | NM_000565.2:993 | Cytokines | Acute-phase response; CD molecules; Cytokines and receptors | | | | |  |  |  |  |  |  |
| IL6ST | NM_002184.2:2505 | Chemokines | CD molecules; Inflammatory response; Chemokines and receptors | | | | | |  |  |  |  |  |
| IL7 | NM_000880.2:38 | Interleukins | Adaptive immune response; Humoral immune response; Interleukins | | | | | |  |  |  |  |  |
| IL7R | NM_002185.2:1610 | Cytokines | Adaptive immune response; CD molecules; Cytokines and receptors | | | | | |  |  |  |  |  |
| IL9 | NM_000590.1:300 | Cytokines | Cytokines and receptors; Interleukins; Regulation of inflammatory response | | | | | |  |  |  |  |  |
| ILF3 | NM_001137673.1:730 | Chemokines | Chemokines and receptors | | |  |  |  |  |  |  |  |  |
| INPP5D | NM_005541.3:4075 | Regulation | Negative regulation of immune response | | | |  |  |  |  |  |  |  |
| IRAK1 | NM_001569.3:1995 | | Innate immune response | |  |  |  |  |  |  |  |  |  |
| IRAK2 | NM_001570.3:1285 | | Innate immune response | |  |  |  |  |  |  |  |  |  |
| IRAK4 | NM_016123.1:2175 | | Innate immune response | |  |  |  |  |  |  |  |  |  |
| IRF1 | NM_002198.1:510 | Chemokines; Regulation; T-Cell Functions | Cytokines and receptors; Th1 orientation; Transcription factors; Transcriptional regulators | | | | | | |  |  |  |  |
| IRF2 | NM_002199.3:1624 | Chemokines; Regulation | Cytokines and receptors; Transcription factors; Transcriptional regulators | | | | | |  |  |  |  |  |
| IRF3 | NM_001571.5:1303 | | Innate immune response | |  |  |  |  |  |  |  |  |  |
| IRF4 | NM_002460.1:325 | Regulation; T-Cell Functions | Regulators of T-cell activation; Transcription factors; Transcriptional regulators; T-cell differentiation | | | | | | | |  |  |  |
| IRF5 | NM_002200.3:1845 | Senescence | Senescence initiators interferon related | | | |  |  |  |  |  |  |  |
| IRF7 | NM_001572.3:1763 | | Innate immune response | |  |  |  |  |  |  |  |  |  |
| IRF8 | NM_002163.2:253 | Chemokines; Regulation | Cytokines and receptors; Transcription factors; Transcriptional regulators | | | | | |  |  |  |  |  |
| IRGM | NM_001145805.1:1412 | | Innate immune response | |  |  |  |  |  |  |  |  |  |
| ISG15 | NM_005101.3:305 | | Innate immune response | |  |  |  |  |  |  |  |  |  |
| ISG20 | NM_002201.4:358 | | Innate immune response | |  |  |  |  |  |  |  |  |  |
| ITCH | NM_001257138.1:438 | | Innate immune response | |  |  |  |  |  |  |  |  |  |
| ITGA1 | NM_181501.1:1875 | Adhesion; T-Cell Functions | Adhesion; CD molecules; T-cell anergy | | |  |  |  |  |  |  |  |  |
| ITGA2 | NM_002203.2:475 | Adhesion | Adhesion; CD molecules | |  |  |  |  |  |  |  |  |  |
| ITGA2B | NM_000419.3:740 | Adhesion | Adhesion; CD molecules | |  |  |  |  |  |  |  |  |  |
| ITGA4 | NM_000885.4:975 | Adhesion; Regulation | Adhesion; CD molecules; Regulation of immune response | | | | |  |  |  |  |  |  |
| ITGA5 | NM_002205.2:925 | Adhesion | Adhesion; CD molecules; Innate immune response | | | |  |  |  |  |  |  |  |
| ITGA6 | NM_000210.1:3065 | Adhesion | Adhesion; CD molecules | |  |  |  |  |  |  |  |  |  |
| ITGAE | NM_002208.4:3405 | Adhesion | Adhesion; CD molecules | |  |  |  |  |  |  |  |  |  |
| ITGAL | NM_002209.2:3905 | Adhesion; Regulation | Adhesion; CD molecules; Regulation of immune response | | | | |  |  |  |  |  |  |
| ITGAM | NM_000632.3:515 | Adhesion; Transporter Functions | Adhesion; CD molecules; Innate immune response; Receptors involved in phagocytosis | | | | | | |  |  |  |  |
| ITGAX | NM_000887.3:700 | Adhesion | Adhesion; CD molecules; Innate immune response | | | |  |  |  |  |  |  |  |
| ITGB1 | NM_033666.2:2000 | Adhesion; Regulation | Adhesion; CD molecules; Regulation of immune response | | | | |  |  |  |  |  |  |
| ITGB2 | NM_000211.2:520 | Adhesion; Regulation | Adhesion; CD molecules; Regulation of immune response | | | | |  |  |  |  |  |  |
| ITGB3 | NM_000212.2:4485 | Adhesion | Adhesion; CD molecules | |  |  |  |  |  |  |  |  |  |
| ITGB4 | NM_001005731.1:4151 | Adhesion | Adhesion; CD molecules | |  |  |  |  |  |  |  |  |  |
| ITK | NM_005546.3:3430 | | Adaptive immune response | | |  |  |  |  |  |  |  |  |
| JAK1 | NM_002227.1:285 | Cytokines; Regulation | Cytokines and receptors; Innate immune response; Positive regulation of immune response | | | | | | |  |  |  |  |
| JAK2 | NM_004972.2:455 | Cytokines; Regulation | Cytokines and receptors; Innate immune response; Positive regulation of immune response | | | | | | |  |  |  |  |
| JAK3 | NM_000215.2:1715 | Cytokines; Regulation | Cytokines and receptors; Innate immune response; Positive regulation of immune response | | | | | | |  |  |  |  |
| JAM3 | NM_032801.3:890 | | Adaptive immune response | | |  |  |  |  |  |  |  |  |
| KIR2DL1 | NM_014218.2:872 | NK Cell Functions; Regulation | CD molecules; NK cell functions; Regulation of immune response | | | | |  |  |  |  |  |  |
| KIR2DL3 | NM_014511.3:592 | NK Cell Functions; Regulation | CD molecules; NK cell functions; Regulation of immune response | | | | |  |  |  |  |  |  |
| KIR2DS1 | NM_014512.1:718 | NK Cell Functions; Regulation | CD molecules; NK cell functions; Regulation of immune response | | | | |  |  |  |  |  |  |
| KIR3DL2 | NM_006737.2:884 | NK Cell Functions; Regulation | CD molecules; NK cell functions; Regulation of immune response | | | | |  |  |  |  |  |  |
| KIR3DL3 | NM_153443.3:539 | NK Cell Functions | CD molecules; NK cell functions | | |  |  |  |  |  |  |  |  |
| KIR3DS1 | NM_001083539.1:1146 | NK Cell Functions; Regulation | CD molecules; NK cell functions; Regulation of immune response | | | | |  |  |  |  |  |  |
| KLRB1 | NM_002258.2:85 | Chemokines; NK Cell Functions | CD molecules; Chemokines and receptors; Inflammatory response; NK cell functions | | | | | | |  |  |  |  |
| KLRC1 | NM_002259.3:335 | NK Cell Functions; Regulation | CD molecules; NK cell functions; Regulation of immune response | | | | |  |  |  |  |  |  |
| KLRC2 | NM_002260.3:942 | NK Cell Functions | CD molecules; NK cell functions | | |  |  |  |  |  |  |  |  |
| KLRG1 | NM_005810.3:65 | NK Cell Functions; Regulation | Innate immune response; NK cell functions; Regulation of immune response | | | | | |  |  |  |  |  |
| KLRK1 | NM_007360.3:522 | NK Cell Functions; Regulation | CD molecules; NK cell functions; Regulation of immune response | | | | |  |  |  |  |  |  |
| LAMP1 | NM_005561.3:2070 | Transporter Functions | Autophagy induction by intracellular pathogens; CD molecules; Genes linking autophagosome to lysosome | | | | | | | | |  |  |
| LAMP2 | NM_001122606.1:46 | | CD molecules | |  |  |  |  |  |  |  |  |  |
| LBP | NM_004139.2:792 | Macrophage Functions | Innate immune response; Macrophage activation | | | |  |  |  |  |  |  |  |
| LCK | NM_005356.2:1260 | Regulation; T-Cell Functions | Regulators of T-cell activation | | |  |  |  |  |  |  |  |  |
| LCN2 | NM_005564.3:325 | | Innate immune response | |  |  |  |  |  |  |  |  |  |
| LCP1 | NM_002298.4:3195 | T-Cell Functions | T-cell activation | |  |  |  |  |  |  |  |  |  |
| LGALS3 | NM_001177388.1:495 | | Innate immune response | |  |  |  |  |  |  |  |  |  |
| LIF | NM_002309.3:1240 | Cell Functions | Basic cell functions | |  |  |  |  |  |  |  |  |  |
| LILRA1 | NM_006863.1:1719 | Regulation | CD molecules; Regulation of immune response | | | |  |  |  |  |  |  |  |
| LILRA4 | NM_012276.3:1577 | | CD molecules; Innate immune response | | | |  |  |  |  |  |  |  |
| LILRA5 | NM_181879.2:545 | | CD molecules; Innate immune response | | | |  |  |  |  |  |  |  |
| LILRB1 | NM_001081637.1:2332 | Regulation; T-Cell Functions | CD molecules; Regulation of immune response; T-cell proliferation | | | | | |  |  |  |  |  |
| LILRB2 | NM_005874.1:595 | Regulation | CD molecules; Regulation of immune response | | | |  |  |  |  |  |  |  |
| LILRB3 | NM_006864.2:2235 | Regulation | CD molecules; Regulation of immune response | | | |  |  |  |  |  |  |  |
| LRP1 | NM_002332.2:4240 | | CD molecules | |  |  |  |  |  |  |  |  |  |
| LRRN3 | NM_001099660.1:2940 | | CD molecules | |  |  |  |  |  |  |  |  |  |
| LTB | NM_002341.1:330 | Cytokines; TNF Superfamily | Cytokines and receptors; TNF superfamily members and their receptors | | | | | |  |  |  |  |  |
| LTBR | NM_002342.1:1435 | Chemokines | Chemokines and receptors | | |  |  |  |  |  |  |  |  |
| LTF | NM_002343.2:590 | | Humoral immune response | | |  |  |  |  |  |  |  |  |
| LY86 | NM_004271.3:255 | | Innate immune response; Humoral immune response | | | | |  |  |  |  |  |  |
| LY9 | NM_001033667.1:260 | | Adaptive immune response; CD molecules | | | |  |  |  |  |  |  |  |
| LY96 | NM_015364.2:360 | | Innate immune response | |  |  |  |  |  |  |  |  |  |
| LYN | NM_002350.1:1285 | Regulation | Negative regulation of immune response; Innate immune response | | | | | |  |  |  |  |  |
| MAP2K1 | NM_002755.2:970 | | Innate immune response | |  |  |  |  |  |  |  |  |  |
| MAP2K2 | NM_030662.2:1325 | | Innate immune response | |  |  |  |  |  |  |  |  |  |
| MAP2K4 | NM_003010.2:2830 | | Innate immune response | |  |  |  |  |  |  |  |  |  |
| MAP3K1 | NM_005921.1:2525 | | Innate immune response | |  |  |  |  |  |  |  |  |  |
| MAP3K5 | NM_005923.3:1760 | | Innate immune response | |  |  |  |  |  |  |  |  |  |
| MAP3K7 | NM_145333.1:670 | | Innate immune response | |  |  |  |  |  |  |  |  |  |
| MAP4K2 | NM_004579.2:1610 | | Innate immune response | |  |  |  |  |  |  |  |  |  |
| MAPK1 | NM_138957.2:430 | | Innate immune response | |  |  |  |  |  |  |  |  |  |
| MAPK11 | NM_002751.5:1310 | | Innate immune response | |  |  |  |  |  |  |  |  |  |
| MAPK14 | NM_001315.1:450 | | Innate immune response | |  |  |  |  |  |  |  |  |  |
| MAPK3 | NM_001040056.1:580 | | Innate immune response | |  |  |  |  |  |  |  |  |  |
| MAPK8 | NM_002750.2:945 | | Innate immune response | |  |  |  |  |  |  |  |  |  |
| MAPKAPK2 | NM_004759.3:710 | | Innate immune response | |  |  |  |  |  |  |  |  |  |
| MASP1 | NM_139125.3:35 | | Innate immune response | |  |  |  |  |  |  |  |  |  |
| MASP2 | NM_139208.1:330 | | Innate immune response | |  |  |  |  |  |  |  |  |  |
| MAVS | NM_020746.3:3460 | | Innate immune response | |  |  |  |  |  |  |  |  |  |
| MBL2 | NM_000242.2:1756 | | Innate immune response | |  |  |  |  |  |  |  |  |  |
| MCAM | NM_006500.2:1515 | Adhesion | Adhesion; CD molecules | |  |  |  |  |  |  |  |  |  |
| MEF2C | NM_002397.3:2445 | | Humoral immune response | | |  |  |  |  |  |  |  |  |
| MEFV | NM_000243.2:1162 | | Innate immune response | |  |  |  |  |  |  |  |  |  |
| MERTK | NM_006343.2:665 | Transporter Functions | Phagyocytosis signal transduction | | |  |  |  |  |  |  |  |  |
| MFGE8 | NM_001114614.1:328 | Transporter Functions | Receptors involved in phagocytosis | | |  |  |  |  |  |  |  |  |
| MICA | NM_000247.1:550 | Regulation | Immune response to tumor cell; Regulation of immune response | | | | |  |  |  |  |  |  |
| MICB | NM_005931.3:1387 | Regulation | Regulation of immune response | | |  |  |  |  |  |  |  |  |
| MIF | NM_002415.1:319 | | Innate immune response | |  |  |  |  |  |  |  |  |  |
| MNX1 | NM_005515.3:1680 | | Humoral immune response | | |  |  |  |  |  |  |  |  |
| MR1 | NM_001531.2:7695 | Antigen Processing | Antigen processing and presentation | | |  |  |  |  |  |  |  |  |
| MRC1 | NM_002438.2:525 | | CD molecules | |  |  |  |  |  |  |  |  |  |
| MST1R | NM_002447.1:3300 | | CD molecules; Innate immune response | | | |  |  |  |  |  |  |  |
| MUC1 | NM_001018017.1:725 | | CD molecules | |  |  |  |  |  |  |  |  |  |
| MX1 | NM_002462.2:1485 | | Innate immune response | |  |  |  |  |  |  |  |  |  |
| MYD88 | NM_002468.3:2145 | TLR | Innate immune response; Toll-like receptor | | | |  |  |  |  |  |  |  |
| NCAM1 | NM_000615.5:1620 | | CD molecules | |  |  |  |  |  |  |  |  |  |
| NCF4 | NM_000631.4:210 | | Innate immune response | |  |  |  |  |  |  |  |  |  |
| NFATC1 | NM_172389.1:1984 | Regulation | Transcription factors; Transcriptional regulators | | | |  |  |  |  |  |  |  |
| NFATC2 | NM_012340.3:1815 | Regulation | Transcription factors; Transcriptional regulators | | | |  |  |  |  |  |  |  |
| NFATC3 | NM_004555.2:2190 | Regulation | Transcriptional regulators | | |  |  |  |  |  |  |  |  |
| NFKB1 | NM_003998.2:1675 | | Innate immune response | |  |  |  |  |  |  |  |  |  |
| NFKB2 | NM_002502.2:825 | | Innate immune response | |  |  |  |  |  |  |  |  |  |
| NFKBIA | NM_020529.1:945 | | Innate immune response | |  |  |  |  |  |  |  |  |  |
| NLRC5 | NM_032206.4:860 | | Innate immune response | |  |  |  |  |  |  |  |  |  |
| NLRP3 | NM_001079821.2:415 | | Innate immune response | |  |  |  |  |  |  |  |  |  |
| NOD1 | NM_006092.1:3285 | | Innate immune response | |  |  |  |  |  |  |  |  |  |
| NOD2 | NM_022162.1:4080 | Cytokines | Innate immune response; Cytokines and receptors | | | |  |  |  |  |  |  |  |
| NOS2A | NM_153292.1:546 | | Inflammatory response | |  |  |  |  |  |  |  |  |  |
| NOTCH1 | NM_017617.3:735 | Regulation | Transcriptional regulators | | |  |  |  |  |  |  |  |  |
| NRP1 | NM_003873.5:370 | Cell Functions | Basic cell functions; CD molecules | | |  |  |  |  |  |  |  |  |
| NT5E | NM_002526.2:1214 | Transporter Functions | CD molecules; Phagocytosis | | |  |  |  |  |  |  |  |  |
| OSM | NM_020530.4:580 | Cell Functions | Basic cell functions | |  |  |  |  |  |  |  |  |  |
| PAX5 | NM_016734.1:2288 | | Humoral immune response | | |  |  |  |  |  |  |  |  |
| PDCD1LG2 | NM_025239.3:235 | | Adaptive immune response; CD molecules | | | |  |  |  |  |  |  |  |
| PDGFC | NM_016205.2:1632 | Cell Functions | Basic cell functions | |  |  |  |  |  |  |  |  |  |
| PDGFRB | NM_002609.3:840 | | CD molecules | |  |  |  |  |  |  |  |  |  |
| PECAM1 | NM_000442.3:1365 | Transporter Functions | CD molecules; Receptors involved in phagocytosis | | | |  |  |  |  |  |  |  |
| PIK3CD | NM_005026.3:2978 | | Adaptive immune response; Innate immune response | | | | |  |  |  |  |  |  |
| PIK3CG | NM_002649.2:2125 | | Adaptive immune response; Innate immune response | | | | |  |  |  |  |  |  |
| PIN1 | NM_006221.2:434 | | Innate immune response | |  |  |  |  |  |  |  |  |  |
| PLA2G1B | NM_000928.2:396 | Regulation | Positive regulation of immune response | | | |  |  |  |  |  |  |  |
| PLAU | NM_002658.2:793 | Senescence | Senescence pathway | |  |  |  |  |  |  |  |  |  |
| PLAUR | NM_001005376.1:440 | | CD molecules | |  |  |  |  |  |  |  |  |  |
| PNMA1 | NM_006029.4:1565 | | Inflammatory response to antigenic stimulus | | | |  |  |  |  |  |  |  |
| POU2AF1 | NM_006235.2:1675 | | Humoral immune response | | |  |  |  |  |  |  |  |  |
| POU2F2 | NM_002698.2:908 | | Humoral immune response | | |  |  |  |  |  |  |  |  |
| PPARG | NM_015869.3:1035 | | Innate immune response | |  |  |  |  |  |  |  |  |  |
| PPBP | NM_002704.2:330 | Chemokines | Chemokines and receptors | | |  |  |  |  |  |  |  |  |
| PRKCD | NM_006254.3:2165 | Senescence | Senescence initiators | |  |  |  |  |  |  |  |  |  |
| PRKCE | NM_005400.2:1695 | Macrophage Functions | Macrophage activation | |  |  |  |  |  |  |  |  |  |
| PSEN1 | NM_000021.2:900 | T-Cell Functions | T-cell activation | |  |  |  |  |  |  |  |  |  |
| PSEN2 | NM_000447.2:915 | T-Cell Functions | T-cell activation | |  |  |  |  |  |  |  |  |  |
| PSMB10 | NM_002801.2:221 | | Humoral immune response | | |  |  |  |  |  |  |  |  |
| PSMB7 | NM_002799.2:420 | Antigen Processing | Antigen processing and presentation | | |  |  |  |  |  |  |  |  |
| PSMB8 | NM_004159.4:1215 | Chemokines | Chemokines and receptors | | |  |  |  |  |  |  |  |  |
| PSMB9 | NM_002800.4:455 | Antigen Processing | Adaptive immune response; Antigen processing and presentation | | | | |  |  |  |  |  |  |
| PSMD7 | NM_002811.3:580 | | CD molecules | |  |  |  |  |  |  |  |  |  |
| PTGS2 | NM_000963.1:495 | Cytokines | Acute-phase response; Cytokines and receptors | | | |  |  |  |  |  |  |  |
| PTPRC | NM_080921.3:258 | B-Cell Functions; T-Cell Functions | B-cell proliferation; CD molecules; T-cell differentiation | | | | |  |  |  |  |  |  |
| PVR | NM_006505.3:604 | Regulation | CD molecules; Regulation of immune response | | | |  |  |  |  |  |  |  |
| PYCARD | NM_013258.3:714 | | Innate immune response | |  |  |  |  |  |  |  |  |  |
| RAG1 | NM_000448.2:2300 | B-Cell Functions | Adaptive immune response; B-cell differentiation | | | |  |  |  |  |  |  |  |
| REL | NM_002908.2:225 | Regulation | Transcription factors | |  |  |  |  |  |  |  |  |  |
| RELA | NM_021975.2:360 | | Innate immune response | |  |  |  |  |  |  |  |  |  |
| RELB | NM_006509.2:250 | Regulation | Transcription factors | |  |  |  |  |  |  |  |  |  |
| RIPK2 | NM_003821.5:1695 | | Innate immune response | |  |  |  |  |  |  |  |  |  |
| RORA | NM_134261.2:1715 | Regulation | Transcription factors | |  |  |  |  |  |  |  |  |  |
| RPS6 | NM_001010.2:171 | T-Cell Functions | T-cell proliferation | |  |  |  |  |  |  |  |  |  |
| RUNX1 | NM_001754.4:635 | Regulation | Transcription factors | |  |  |  |  |  |  |  |  |  |
| RUNX3 | NM_004350.1:2085 | Regulation | Transcription factors | |  |  |  |  |  |  |  |  |  |
| S100A12 | NM_005621.1:260 | | Innate immune response | |  |  |  |  |  |  |  |  |  |
| S100A7 | NM_002963.2:69 | | Innate immune response | |  |  |  |  |  |  |  |  |  |
| S100A8 | NM_002964.3:115 | | Inflammatory response | |  |  |  |  |  |  |  |  |  |
| S100B | NM_006272.1:85 | | Innate immune response | |  |  |  |  |  |  |  |  |  |
| SAA1 | NM_199161.1:135 | | Innate immune response | |  |  |  |  |  |  |  |  |  |
| SBNO2 | NM_014963.2:2002 | Macrophage Functions | Macrophage activation | |  |  |  |  |  |  |  |  |  |
| SELE | NM_000450.2:1505 | Regulation | CD molecules; Regulation of inflammatory response | | | |  |  |  |  |  |  |  |
| SELL | NR_029467.1:1585 | Regulation | CD molecules; Regulation of immune response | | | |  |  |  |  |  |  |  |
| SELPLG | NM_001206609.1:326 | | CD molecules | |  |  |  |  |  |  |  |  |  |
| SERPINB2 | NM_002575.1:305 | Senescence | Senescence initiators interferon related | | | |  |  |  |  |  |  |  |
| SERPING1 | NM_000062.2:305 | | Innate immune response | |  |  |  |  |  |  |  |  |  |
| SH2B2 | NM_020979.3:1567 | Regulation | Regulation of immune response | | |  |  |  |  |  |  |  |  |
| SH2D1A | NM_001114937.2:495 | | Humoral immune response | | |  |  |  |  |  |  |  |  |
| SH2D1B | NM_053282.4:545 | Leukocyte Functions | Leukocyte activation | |  |  |  |  |  |  |  |  |  |
| SIGIRR | NM_021805.2:469 | | Innate immune response | |  |  |  |  |  |  |  |  |  |
| SIGLEC1 | NM_023068.3:5165 | Transporter Functions | CD molecules; Phagocytosis recognition and engulfment | | | | |  |  |  |  |  |  |
| SLAMF1 | NM_003037.2:580 | | CD molecules; Adaptive immune response | | | |  |  |  |  |  |  |  |
| SLAMF6 | NM_001184714.1:1032 | | CD molecules | |  |  |  |  |  |  |  |  |  |
| SLAMF7 | NM_021181.3:215 | | CD molecules; Innate immune response | | | |  |  |  |  |  |  |  |
| SLC11A1 | NM_000578.2:1965 | T-Cell Functions | T-cell proliferation | |  |  |  |  |  |  |  |  |  |
| SMAD3 | NM_005902.3:4220 | Regulation | Regulation of immune response | | |  |  |  |  |  |  |  |  |
| SOCS1 | NM_003745.1:1025 | T-Cell Functions | T-cell differentiation | |  |  |  |  |  |  |  |  |  |
| SPINK5 | NM_006846.3:2595 | Regulation | Negative regulation of immune response | | | |  |  |  |  |  |  |  |
| SPN | NM_003123.3:2345 | Regulation | CD molecules; Regulation of immune response | | | |  |  |  |  |  |  |  |
| SPP1 | NM_000582.2:760 | Cytokines | Cytokines and receptors | |  |  |  |  |  |  |  |  |  |
| ST6GAL1 | NM_003032.2:1300 | | Humoral immune response | | |  |  |  |  |  |  |  |  |
| STAT1 | NM_007315.2:205 | Chemokines; Regulation | Cytokines and receptors; Transcription factors; Transcriptional regulators | | | | | |  |  |  |  |  |
| STAT2 | NM_005419.2:1965 | Chemokines; Regulation | Cytokines and receptors; Transcription factors; Transcriptional regulators | | | | | |  |  |  |  |  |
| STAT3 | NM_139276.2:4535 | Chemokines; Regulation | Cytokines and receptors; Transcription factors; Transcriptional regulators | | | | | |  |  |  |  |  |
| STAT5B | NM_012448.3:200 | Chemokines; Regulation | Adaptive immune response; Cytokines and receptors; Transcription factors; Transcriptional regulators | | | | | | | |  |  |  |
| SYK | NM_003177.3:1685 | Macrophage Functions | Macrophage activation; Adaptive immune response; Innate immune response | | | | | |  |  |  |  |  |
| TAB1 | NM_153497.2:614 | | Innate immune response | |  |  |  |  |  |  |  |  |  |
| TAL1 | NM_003189.2:4635 | Regulation | Transcription factors; Adaptive immune response | | | |  |  |  |  |  |  |  |
| TANK | NM_004180.2:110 | | Innate immune response | |  |  |  |  |  |  |  |  |  |
| TAP1 | NM_000593.5:2075 | Antigen Processing | Adaptive immune response; Antigen processing and presentation | | | | |  |  |  |  |  |  |
| TAP2 | NM_000544.3:909 | Antigen Processing | Adaptive immune response; Antigen processing and presentation | | | | |  |  |  |  |  |  |
| TAPBP | NM_003190.4:1536 | Antigen Processing | Adaptive immune response; Antigen processing and presentation | | | | |  |  |  |  |  |  |
| TBK1 | NM_013254.2:1610 | | Innate immune response | |  |  |  |  |  |  |  |  |  |
| TCF7 | NM_003202.2:2420 | Regulation | Transcription factors | |  |  |  |  |  |  |  |  |  |
| TFE3 | NM_006521.3:2935 | | Humoral immune response | | |  |  |  |  |  |  |  |  |
| TFEB | NM_007162.2:2026 | | Humoral immune response | | |  |  |  |  |  |  |  |  |
| TFRC | NM_003234.1:1220 | | CD molecules | |  |  |  |  |  |  |  |  |  |
| TGFB1 | NM_000660.3:1260 | Interleukins; Regulation | Immunosuppression; Interleukins; Negative regulation of immune response | | | | | |  |  |  |  |  |
| TGFB2 | NM_003238.2:1125 | Interleukins | Immunosuppression; Interleukins | | |  |  |  |  |  |  |  |  |
| THBD | NM_000361.2:1246 | Leukocyte Functions | CD molecules; Leukocyte migration | | |  |  |  |  |  |  |  |  |
| THY1 | NM_006288.2:135 | | CD molecules | |  |  |  |  |  |  |  |  |  |
| TICAM1 | NM_014261.1:518 | Macrophage Functions | Macrophage activation; Innate immune response | | | |  |  |  |  |  |  |  |
| TICAM2 | NM_021649.4:3234 | | Innate immune response | |  |  |  |  |  |  |  |  |  |
| TIGIT | NM_173799.2:1968 | T-Cell Functions | Adaptive immune response; T-cell activation | | | |  |  |  |  |  |  |  |
| TIRAP | NM_148910.2:661 | | Innate immune response | |  |  |  |  |  |  |  |  |  |
| TLR1 | NM_003263.3:545 | Microglial Functions; TLR | CD molecules; Innate immune response; Microglial cell activation; Toll-like receptor | | | | | | |  |  |  |  |
| TLR10 | NM_030956.2:2246 | TLR | CD molecules; Innate immune response; Toll-like receptor | | | | |  |  |  |  |  |  |
| TLR2 | NM_003264.3:180 | TLR | CD molecules; Innate immune response; Toll-like receptor | | | | |  |  |  |  |  |  |
| TLR3 | NM_003265.2:230 | Microglial Functions; TLR | CD molecules; Innate immune response; Microglial cell activation; Toll-like receptor | | | | | | |  |  |  |  |
| TLR4 | NM_138554.2:2570 | Microglial Functions; TLR | CD molecules; Innate immune response; Microglial cell activation; Toll-like receptor | | | | | | |  |  |  |  |
| TLR5 | NM_003268.3:215 | TLR | Innate immune response; Toll-like receptor | | | |  |  |  |  |  |  |  |
| TLR6 | NM_006068.2:2530 | TLR | CD molecules; Innate immune response; Toll-like receptor | | | | |  |  |  |  |  |  |
| TLR7 | NM_016562.3:4120 | Microglial Functions; TLR | Microglial cell activation; Innate immune response; Toll-like receptor | | | | | |  |  |  |  |  |
| TLR8 | NM_016610.2:2310 | TLR | CD molecules; Innate immune response; Toll-like receptor | | | | |  |  |  |  |  |  |
| TLR9 | NM_017442.2:985 | TLR | CD molecules; Innate immune response; Toll-like receptor | | | | |  |  |  |  |  |  |
| TNF | NM_000594.2:1010 | Interleukins; TNF Superfamily | Humoral immune response; Interleukins; TNF superfamily members and their receptors | | | | | | |  |  |  |  |
| TNFAIP3 | NM_006290.2:260 | TNF Superfamily | Innate immune response; TNF superfamily members and their receptors | | | | | |  |  |  |  |  |
| TNFRSF10B | NM_003842.3:565 | TNF Superfamily | CD molecules; TNF superfamily members and their receptors | | | | |  |  |  |  |  |  |
| TNFRSF10C | NM_003841.3:682 | TNF Superfamily | CD molecules; TNF superfamily members and their receptors | | | | |  |  |  |  |  |  |
| TNFRSF11A | NM_003839.2:490 | TNF Superfamily | Adaptive immune response; CD molecules; TNF superfamily members and their receptors | | | | | | |  |  |  |  |
| TNFRSF11B | NM_002546.2:1075 | TNF Superfamily | TNF superfamily members and their receptors | | | |  |  |  |  |  |  |  |
| TNFRSF12A | NM_016639.1:575 | TNF Superfamily | CD molecules; TNF superfamily members and their receptors | | | | |  |  |  |  |  |  |
| TNFRSF13B | NM_012452.2:160 | Chemokines; TNF Superfamily | CD molecules; Chemokines and receptors; TNF superfamily members and their receptors | | | | | | |  |  |  |  |
| TNFRSF13C | NM_052945.3:789 | Regulation; TNF Superfamily | CD molecules; Regulation of immune response; TNF superfamily members and their receptors | | | | | | | |  |  |  |
| TNFRSF14 | NM_003820.2:916 | Regulation; T-Cell Functions; TNF Superfamily | CD molecules; T-cell regulators; TNF superfamily members and their receptors | | | | | |  |  |  |  |  |
| TNFRSF18 | NM_004195.2:445 | TNF Superfamily | TNF superfamily members and their receptors | | | |  |  |  |  |  |  |  |
| TNFRSF1A | NM_001065.2:515 | Chemokines; TNF Superfamily | CD molecules; Chemokines and receptors; TNF superfamily members and their receptors | | | | | | |  |  |  |  |
| TNFRSF1B | NM_001066.2:835 | Chemokines; TNF Superfamily | CD molecules; Chemokines and receptors; TNF superfamily members and their receptors | | | | | | |  |  |  |  |
| TNFRSF8 | NM_152942.2:2030 | TNF Superfamily | CD molecules; TNF superfamily members and their receptors | | | | |  |  |  |  |  |  |
| TNFSF10 | NM_003810.2:115 | Cytokines; Cell Cycle; Regulation; TNF Superfamily | CD molecules; Co-Regulators of autophagy and apoptosis/cell cycle; Cytokines and receptors; TNF superfamily members and their receptors | | | | | | | | | | |
| TNFSF11 | NM_003701.2:490 | Transporter Functions; TNF Superfamily | CD molecules; Phagosome processing; TNF superfamily members and their receptors | | | | | | |  |  |  |  |
| TNFSF12 | NM_003809.2:339 | Chemokines; TNF Superfamily | Chemokines and receptors; TNF superfamily members and their receptors | | | | | |  |  |  |  |  |
| TNFSF13 | NM_003808.3:810 | Regulation; TNF Superfamily | CD molecules; Regulation of immune response; TNF superfamily members and their receptors | | | | | | | |  |  |  |
| TNFSF13B | NM_006573.4:1430 | Regulation; TNF Superfamily | CD molecules; Regulation of immune response; TNF superfamily members and their receptors | | | | | | | |  |  |  |
| TNFSF14 | NM_003807.3:612 | Cytokines; Regulation; T-Cell Functions; TNF Superfamily | CD molecules; Cytokines and receptors; Regulators of T-cell activation; TNF superfamily members and their receptors; T-cell regulators; T-cell proliferation | | | | | | | | | | |
| TNFSF15 | NM_001204344.1:2338 | Chemokines; TNF Superfamily | Chemokines and receptors; TNF superfamily members and their receptors | | | | | |  |  |  |  |  |
| TNFSF18 | NM_005092.2:175 | T-Cell Functions; TNF Superfamily | T-cell proliferation; TNF superfamily members and their receptors | | | | | |  |  |  |  |  |
| TNFSF4 | NM_003326.2:545 | Chemokines; TNF Superfamily | CD molecules; Chemokines and receptors; TNF superfamily members and their receptors | | | | | | |  |  |  |  |
| TNFSF8 | NM_001244.3:518 | Cytokines; TNF Superfamily | CD molecules; Cytokines and receptors; TNF superfamily members and their receptors | | | | | | |  |  |  |  |
| TOLLIP | NM_019009.2:1320 | | Innate immune response | |  |  |  |  |  |  |  |  |  |
| TP53 | NM_000546.2:1330 | T-Cell Functions | T-cell proliferation | |  |  |  |  |  |  |  |  |  |
| TRAF2 | NM_021138.3:1325 | | Innate immune response | |  |  |  |  |  |  |  |  |  |
| TRAF3 | NM_145725.1:1795 | | Innate immune response | |  |  |  |  |  |  |  |  |  |
| TRAF6 | NM_145803.1:1839 | | Innate immune response | |  |  |  |  |  |  |  |  |  |
| TREM1 | NM_018643.3:375 | | CD molecules; Humoral immune response | | | |  |  |  |  |  |  |  |
| TREM2 | NM_018965.3:611 | | Humoral immune response | | |  |  |  |  |  |  |  |  |
| TXK | NM_003328.1:800 | | Adaptive immune response | | |  |  |  |  |  |  |  |  |
| TXNIP | NM_006472.1:255 | | Innate immune response | |  |  |  |  |  |  |  |  |  |
| TYK2 | NM_003331.3:485 | Cytokines; Regulation; Pathogen Defense | Cytokines and receptors; Defense response to virus; Innate immune response; Positive regulation of immune response | | | | | | | | |  |  |
| UBC | NM_021009.3:1875 | | Innate immune response | |  |  |  |  |  |  |  |  |  |
| ULBP2 | NM_025217.2:905 | Regulation | Regulation of immune response | | |  |  |  |  |  |  |  |  |
| VCAM1 | NM_001078.3:2535 | Adhesion; Regulation | Adhesion; CD molecules; Regulation of immune response | | | | |  |  |  |  |  |  |
| VEGFA | NM_001025366.1:1325 | Cytokines; Leukocyte Functions | Leukocyte migration; Cytokines and receptors | | | |  |  |  |  |  |  |  |
| VEGFC | NM_005429.2:565 | | Response to drug | |  |  |  |  |  |  |  |  |  |
| XCL2 | NM_003175.3:377 | Chemokines | Chemokines and receptors | | |  |  |  |  |  |  |  |  |
| XCR1 | NM_005283.2:700 | Chemokines | Chemokines and receptors; Inflammatory response | | | |  |  |  |  |  |  |  |
| YTHDF2 | NM_001172828.1:275 | | Humoral immune response | | |  |  |  |  |  |  |  |  |
| ZAP70 | NM_001079.3:1175 | | Adaptive immune response | | |  |  |  |  |  |  |  |  |
| ADORA2A | NM_000675.3:1095 | Cell Functions | Basic cell functions | |  |  |  |  |  |  |  |  |  |
| BTLA | NM_181780.2:305 | Cell Functions | Basic cell functions; CD molecules | | |  |  |  |  |  |  |  |  |
| CD27 | NM_001242.4:330 | B-Cell Functions | B-cell differentiation; CD molecules | | |  |  |  |  |  |  |  |  |
| CD40LG | NM_000074.2:1225 | Regulation | Adaptive immune response; CD molecules; Regulation of immune response | | | | | |  |  |  |  |  |
| HAVCR2 | NM_032782.3:955 | Cell Functions | Basic cell functions | |  |  |  |  |  |  |  |  |  |
| KIR3DL1 | NM_013289.2:1691 | NK Cell Functions; Regulation | CD molecules; NK cell functions; Regulation of immune response | | | | |  |  |  |  |  |  |
| LAG3 | NM_002286.5:1735 | Regulation; T-Cell Functions | Adaptive immune response; CD molecules; Negative regulation of immune response; T-cell activation | | | | | | | |  |  |  |
| TNFRSF4 | NM_003327.2:200 | TNF Superfamily | CD molecules; TNF superfamily members and their receptors | | | | |  |  |  |  |  |  |
| TNFRSF9 | NM_001561.4:255 | TNF Superfamily | CD molecules; TNF superfamily members and their receptors | | | | |  |  |  |  |  |  |
| Â© 2015-2017 NanoString Technologies | | | |  |  |  |  |  |  |  |  |  |  |

**Supplementary Table S1:** Specific transcripts included in Nanostring panel, with HUGO gene nomenclature, probe ID and immune category shown.

**Supplementary Table S2: Nanostring Immune Categories**

| **T-cell** | **CD8 T-cell** | **Cytotoxic cell** | **Th1 cell** | **Tcm** | **Tem** | **T helper cell** | **TFH** | **Th2 cell** | **Th17 cell** | **Tgd** | **Treg** |
| --- | --- | --- | --- | --- | --- | --- | --- | --- | --- | --- | --- |
| CD2 | CD8A | GNLY | CD38 | ATM | AKT3 | ANP32B | BCL6 | CXCR6 | IL17A | CD160 | FOXP3 |
| CD3E | CD8B | GZMA | CSF2 | DOCK9 | CCR2 | BATF | CXCL13 | GATA3 | IL17RA | FEZ1 |  |
| CD3G | FLT3LG | GZMH | IFNG | NEFL | EWSR1 | NUP107 | MAF | IL26 | RORC | TARP |  |
| CD6 | GZMM | KLRD1 | IL12RB2 | REPS1 | LTK | CD28 | PDCD1 | LAIR2 |  |  |  |
|  | PRF1 | KLRF1 | LTA | USP9Y | NFATC4 | ICOS |  | PMCH |  |  |  |
|  |  |  | STAT4 |  |  |  |  | SMAD2 |  |  |  |
|  |  |  | TBX21 |  |  |  |  | STAT6 |  |  |  |
|  |  |  | CTLA4 |  |  |  |  |  |  |  |  |

| **NK cell** | **NK CD56bright cell** | **NK CD56dim cell** | **B-cell** | **Macrophages** | **DC** | **iDC** | **pDC** | **aDC** | **Neutrophils** |
| --- | --- | --- | --- | --- | --- | --- | --- | --- | --- |
| BCL2 | FOXJ1 | GTF3C1 | BLK | APOE | CCL13 | CD1A | IL3RA | CCL1 | CSF3R |
| FUT5 | MPPED1 | GZMB | CD19 | CCL7 | CCL17 | CD1B |  | EBI3 | FPR2 |
| NCR1 | PLA2G6 | IL21R | CR2 | CD68 | CCL22 | CD1E |  | IDO1 | MME |
| ZNF205 | RRAD |  | HLA-DOB | CHIT1 | CD209 | F13A1 |  | LAMP3 |  |
|  |  |  | MS4A1 | CXCL5 | HSD1181 | SYT17 |  | OAS3 |  |
|  |  |  | TNFRSF17 | MARCO |  |  |  |  |  |
|  |  |  |  | MSR1 |  |  |  |  |  |

| **Eosinophils** | **Mast cell** |
| --- | --- |
| CCR3 | CMA1 |
| IL5RA | CTSG |
| PTGDR2 | KIT |
| SMPD3 | MS4A2 |
| THBS1 | PRG2 |
|  | TPSAB1 |
|  |  |

**Supplementary Table S2:** Immune response categories as defined by Nanostring annotations

**Supplementary Table S3: Pathway Enrichment Analysis**

|  | **Post vs pre treatment all samples** | | |
| --- | --- | --- | --- |
| **Category** | **Up regulated genes** | **Down regulated genes** | **available genes** |
| T-Cell Functions(72) | CD8A CD3G | IL13RA1 RPS6 | 72 |
| Chemokines(99) | CMKLR1 STAT2 TNFRSF13B | IL17B ILF3 LTBR | 99 |
| Cell Functions(154) | CD8A MME CD3G CD6 CD38 KLRC1 | F13A1 EWSR1 IL13RA1 RPS6 | 154 |
| Cytokines(56) | IL1R2 NOD2 TNFSF10 | IL13RA1 | 56 |
| Regulation(154) | CD3G CD38 CD247 CDKN1A HLA-C ITGAL KLRC1 SELL STAT2 TNFSF10 | CMA1 CD200 MICA PVR | 154 |
| Antigen Processing(22) | CD8A HLA-C |  | 22 |
| TNF Superfamily(30) | TNFRSF13B TNFSF10 TNFRSF9 |  | 30 |
| Cell Cycle(13) | CDKN1A TNFSF10 |  | 13 |
| Pathogen Defense(12) | CD8A PRF1 |  | 12 |
| Cytotoxicity(10) | PRF1 HLA-C |  | 10 |
|  |  |  |  |
|  |  |  |  |
|  | **Complete responder vs non responders pre-treatment** | | |
| **Category** | **Up regulated genes** | **Down regulated genes** | **available genes** |
| Chemokines | CX3CR1 | LTBR PPBP | 99 |
| Cytokines |  | IL12RB2 IFNL1 | 56 |
| Regulation | C3 FCGR2B HLA-DMA ITGB2 KLRK1 SELL | IL2RA PLA2G1B | 154 |
| T-Cell Functions | CD8A PTPRC TIGIT | IL12RB2 | 72 |
| Cell Functions | CD8A CD68 CHIT1 MSR1 MPPED1 GZMK KLRK1 PTPRC TIGIT HAVCR2 | HSD11B1 IL12RB2 | 154 |
| Interleukins |  | IFNL1 IL23A IL34 | 38 |
| Transporter Functions | CRP ITGAM |  | 22 |
| Adhesion | ITGAM ITGAX ITGB2 |  | 25 |
| Antigen Processing | CD8A HLA-DMA HLA-DPA1 HLA-DPB1 |  | 22 |
|  |  |  |  |
|  | **Complete responder vs non responders post-treatment** | | |
| **Category** | **Up regulated genes** | **Down regulated genes** | **available genes** |
| Cytokines | IL12RB2 CCL5 CCR5 IL7R | PTGS2 | 56 |
| Regulation | KLRD1 STAT4 CCR7 CXCL9 CXCR4 HLA-A HLA-G ICAM2 INPP5D KLRK1 LCK RUNX3 SPINK5 LAG3 | CTSG | 154 |
| TNF Superfamily | TNFRSF1B | TNFRSF11B TNFRSF1A | 30 |
| Chemokines | CXCL13 STAT4 CCL5 CCR7 CSF2RB CXCL9 CXCR3 CXCR4 IL2RG TNFRSF1B | CCL28 LTBR TNFRSF1A | 99 |
| T-Cell Functions | IL12RB2 STAT4 CCR5 CXCL9 CXCR3 CXCR4 IL18RAP LCK TIGIT LAG3 |  | 72 |
| Cell Functions | GZMA KLRD1 SMPD3 GZMB NCR1 IL12RB2 STAT4 CCR5 CXCL9 CXCR3 CXCR4 GZMK IL18RAP KLRC2 KLRK1 LCK TIGIT CD27 LAG3 | CD1A USP9Y KIR3DL3 | 154 |
| NK Cell Functions | KLRD1 NCR1 KLRC2 KLRK1 | KIR3DL3 | 17 |
| Cytotoxicity | GNLY GZMA GZMB GZMK HLA-A |  | 10 |
|  |  |  |  |

**Supplementary Table S3:** Pathway enrichment analysis results shown demonstrating specific genes up-regulated and down-regulated in each pathway by post- vs pre-treatment in all samples, CR vs NR pre-treatment, and CR vs NR post-treatment with imiquimod.
